# Identifying trajectories across care modalities before and after COVID-19 using sequence analysis

**DOI:** 10.1038/s41746-026-02766-4

**Published:** 2026-05-26

**Authors:** Taylor Rapson, Kathryn E. Kemper-McIsaac, Elizabeth B. Sherwin, Namuun Clifford, Lucia Pacca, Anna Rubinsky, Courtney R. Lyles, Elaine C. Khoong

**Affiliations:** 1 Department of Health Systems and Population Health, University of Washington, Seattle, WA USA; 2 Division of General Internal Medicine, Department of Medicine, University of California, San Francisco, San Francisco, CA USA; 3 Department of Epidemiology & Biostatistics, University of California, San Francisco, San Francisco, CA USA; 4 School of Nursing, The University of Texas at Austin, Austin, TX USA; 5 Philip R. Lee Institute for Health Policy Studies, University of California, San Francisco, San Francisco, CA USA; 6 Academic Research Services, Information Technology, University of California, San Francisco, San Francisco, CA USA; 7 Center for Healthcare Policy and Research, University of California at Davis, School of Medicine, Sacramento, CA USA; 8 Action Research Center for Health, Zuckerberg San Francisco General Hospital, University of California, San Francisco, San Francisco, CA USA; 9 Division of Clinical Informatics and Digital Transformation (DoC-IT), Department of Medicine, University of California, San Francisco, San Francisco, CA USA

**Keywords:** Diseases, Health care, Medical research

## Abstract

The COVID-19 pandemic propelled the growth of virtual care, yet its longitudinal impact on care utilization remains understudied. We used multichannel sequence analysis to classify 10,671 adults with diabetes by outpatient utilization patterns with their primary and diabetes care teams, including remote and in-person visits, at two San Francisco health systems from April 2019 to March 2023. Patients who transitioned to digital care were disproportionately Black or White, Medicare beneficiaries, and had higher comorbidity burdens. Individuals who increased their combined in-person and remote healthcare utilization were disproportionately Hispanic, Spanish-preferring, and had greater disease burden. Those who decreased utilization were predominantly Asian, preferred Chinese, and had low patient portal engagement. While technology-enabled care models have the potential to enhance access, some populations may be at risk of reduced access to care. Health systems should consider targeted interventions to ensure equitable telehealth access for populations at risk of digital exclusion as remote care expands.

## Introduction

The COVID-19 pandemic spurred the growth of telehealth (scheduled video and/or telephone encounters) and between-visit care, such as unscheduled telephone visits and secure messaging in the patient portal, as alternative modalities for receiving healthcare^1–3^. Use of digital health technology and personal devices to engage in remote care has been linked to improved quality of care and clinical outcomes, particularly among individuals with chronic disease^4–9^. Evidence suggests that using the patient portal to send secure messages, request refills, and schedule appointments is linked to patient empowerment and engagement in their care, as well as improved patient-provider communication^6–12^. Similarly, use of the patient portal and telehealth has been shown to have a positive effect on self-management behaviors and clinical outcomes across chronic conditions, such as improved symptom control and medication adherence, which are critical drivers of health^4,6–8,13–16^.

Despite the well-documented increase in the use of telehealth visits and between-visit care as additional access points for engagement with primary care clinical teams in recent years, the longitudinal healthcare utilization patterns comprising multiple modalities of care and their differences across sociodemographic groups are understudied. Prior research has documented unequal uptake of digital healthcare across racial and ethnic groups, language preference, age, and gender, as well as differences in use for those facing barriers to accessing broadband internet, devices, or cellular data (e.g., due to cost)^7,10,14,15,17–26^. In particular, older adults, especially those with non-English language preferences or who identify as a racial or ethnic minority, experience significant disparities in digital health adoption due to disparities in device access, cellular data access, or digital literacy^8,18,22,27^. Together, these barriers create a “digital divide” that may worsen inequities in healthcare settings that increasingly rely on digital health tools, especially in systems serving low-income, diverse populations^8,14,21,28,29^. As digital health becomes further embedded in healthcare delivery, these barriers to the uptake and use of digital health tools may ultimately shape healthcare utilization and outcomes for marginalized populations^30^.

These concerns are particularly salient for patients with Type 2 Diabetes Mellitus (T2DM), one of the most common chronic conditions, and one that disproportionately affects marginalized populations. Patients with uncontrolled diabetes and complications are disproportionately represented in minoritized racial and ethnic groups and lower socioeconomic neighborhoods, highlighting the need for equitable care modalities^14,31,32^. Because effective diabetes management requires ongoing monitoring, communication, and care coordination, patients with T2DM may benefit substantially from expanded modalities for accessing care. Remote tools can help address barriers to in-person care, support continuity between visits, and encourage better disease self-management. Yet, given persistent disparities in both digital health adoption and diabetes control, it remains uncertain whether the expansion of telehealth and patient portal use during the pandemic has narrowed or widened existing inequities.

Understanding healthcare utilization patterns is, therefore, critical—not only to evaluate how patients with T2DM and other chronic conditions engage with multiple modalities of care, but also to identify where gaps in care delivery may exacerbate disparities. However, most prior studies rely on repeated cross-sectional data, examining a single care modality and classifying individuals as either “high” or “low” utilizers of care^33–36^. Such designs capture snapshots rather than trajectories, limiting the ability to understand the multifaceted nature of healthcare utilization and how it evolves over time, particularly in an era of expanded digital health use^2–4^. In contrast, a longitudinal, sequence-based approach can reveal how individuals transition between in-person, telehealth, and between-visit modalities, illuminating dynamic patterns that reflect real-world care experiences. Identifying patterns of underutilization across modalities can further pinpoint unmet needs and opportunities to improve equity. For example, a recent study highlighted that when telehealth, between-visit care, and in-person care were available to patients, there were fewer differences in utilization by race and ethnicity and preferred language, suggesting that multiple options for care utilization can reduce disparities^30^. Still, research remains limited on how these alternative access points to care can alleviate barriers to care and interact over time to supplement traditional care-seeking patterns^8,33^.

Sequence analysis is a data-driven approach that compares and groups longitudinal trajectories of events, making it well-suited for evaluating healthcare utilization patterns, as it preserves the timing, order, and duration of event^37–39^. While studies often summarize utilization as aggregate counts over fixed windows, multichannel sequence analysis can reveal when and in what order patients use in-person, telehealth, and between-visit care, identifying recognizable patterns (for example, “transitioned to digital” or “reduced care engagement”)^39–41^. This moves beyond simple categorizations of “high” or “low” utilizers and can surface nuanced variation in utilization patterns. The ability to cluster patients based on the timing, order, and duration of their healthcare encounters can provide a more granular understanding of patient heterogeneity and reveal hidden patterns and subgroups^42,43^.

This study advances existing research by employing multichannel sequence analysis and cluster analysis to create clusters of patients with similar temporal sequences of in-person, between-visit, and telehealth interactions across two health systems. We examine sociodemographic differences between clusters of healthcare utilization to assess whether specific patient characteristics are disproportionately represented in certain clusters. By comparing clusters across two health systems, we aim to (1) identify heterogeneities in multi-modality healthcare utilization over time, (2) assess whether clusters of care utilization are patterned by sociodemographic factors, which could suggest potential inequities in access and utilization, and (3) assess whether care utilization patterns are consistent across health systems or vary by site, potentially reflecting differences in patient populations, resources, and care delivery models. Given the potential role of remote care engagement to either mitigate or perpetuate existing disparities, the evidence from these comparisons can shape policies that promote equitable integration of digital health across diverse healthcare settings.

## Results

First, we separately outlined the visit trajectories within each healthcare system, given their unique patient populations and different uptake of telehealth and between-visit care during and post-pandemic.

### UCSF

After excluding patients without a visit in the last 12 months of the study period, we identified a cohort of 3860 patients with T2DM at UCSF (Table 1). Over half (53%) of patients were aged 65+. Most patients were English-speaking (82%), and the cohort included high proportions of Asian (40%, including 12% with non-English preference) and White (24%) patients. Patients were relatively evenly distributed across nSES quintiles, with 17% residing in the lowest and 18% in the highest quintile. Most patients in the cohort had insurance coverage through Commercial/private (44%) and Medicare plans (43%). Almost a quarter (23%) of patients had high comorbidity scores (CCI 3+), and nearly a third had uncontrolled A1c at baseline. Digital engagement was high, with 78% of patients having an active MyChart account at baseline.

**Table 1:** Patient sociodemographic and clinical characteristics at UCSF and SFHN

	UCSF	SFHN
	*n* = 3860	*n* = 6811
Age category (%)		
18–34	91 (2%)	162 (2%)
35–49	472 (12%)	1080 (16%)
50–64	1234 (32%)	3215 (47%)
65–74	1118 (29%)	1727 (25%)
75+	945 (24%)	627 (9%)
Sex (%)		
Female	2091 (54%)	3581 (53%)
Male	1769 (46%)	3229 (47%)
Joint race eth and language (%)		
Asian and English preference	1050 (27%)	641 (9%)
Asian and non-English preference	478 (12%)	1753 (26%)
Black/African American and English preference	527 (14%)	1027 (15%)
Hispanic and English preference	366 (9%)	449 (7%)
Hispanic and non-English preference	114 (3%)	1881 (28%)
Other/Un and English preference	317 (8%)	254 (4%)
Other/Un and Non-English Preference	67 (2%)	84 (1%)
White and English preference	941 (24%)	722 (11%)
Race or ethnicity (%)		
Asian	1528 (40%)	2394 (35%)
Black/African American	527 (14%)	1027 (15%)
Hispanic/Latino	480 (12%)	2330 (34%)
Other/Unknown	384 (10%)	338 (5%)
White	941 (24%)	722 (11%)
Language preference (%)		
Chinese	296 (8%)	1324 (19%)
English	3154 (82%)	2931 (43%)
Other	298 (8%)	655 (10%)
Spanish	112 (3%)	1901 (28%)
Insurance (%)		
Commercial	1708 (44%)	71 (1%)
Healthy workers	0 (0%)	1049 (15%)
Medicaid	502 (13%)	2709 (40%)
Medicare	1644 (43%)	2126 (31%)
Missing	5 (0%)	28 (0%)
Uninsured	1 (0%)	828 (12%)
Neighborhood SES (1 = lowest) (%)		
1 (lowest)	651 (17%)	2561 (38%)
2	689 (18%)	1771 (26%)
3	752 (19%)	1049 (15%)
4	886 (23%)	889 (13%)
5 (highest)	698 (18%)	405 (6%)
Missing	184 (5%)	136 (2%)
Charlson score (%)		
0–2	2965 (77%)	5818 (85%)
3+	895 (23%)	993 (15%)
Baseline A1c control (%)		
Uncontrolled A1c (≥8)	818 (21%)	2631 (39%)
Controlled A1c (<8)	2611 (68%)	3307 (49%)
Missing	431 (11%)	873 (13%)
Baseline BP control (%)		
BP ≤ 120/80	658 (17%)	1629 (24%)
BP ≤ 140/90	2126 (55%)	3598 (53%)
BP > 140/90	930 (24%)	1205 (18%)
Missing	146 (4%)	379 (6%)
Active MyChart (%)		
No	861 (22%)	6217 (91%)
Yes	2999 (78%)	594 (9%)

*SES* socioeconomic status, *A1c* hemoglobin A1c lab result, *BP* blood pressure.

**Fig. 1:**
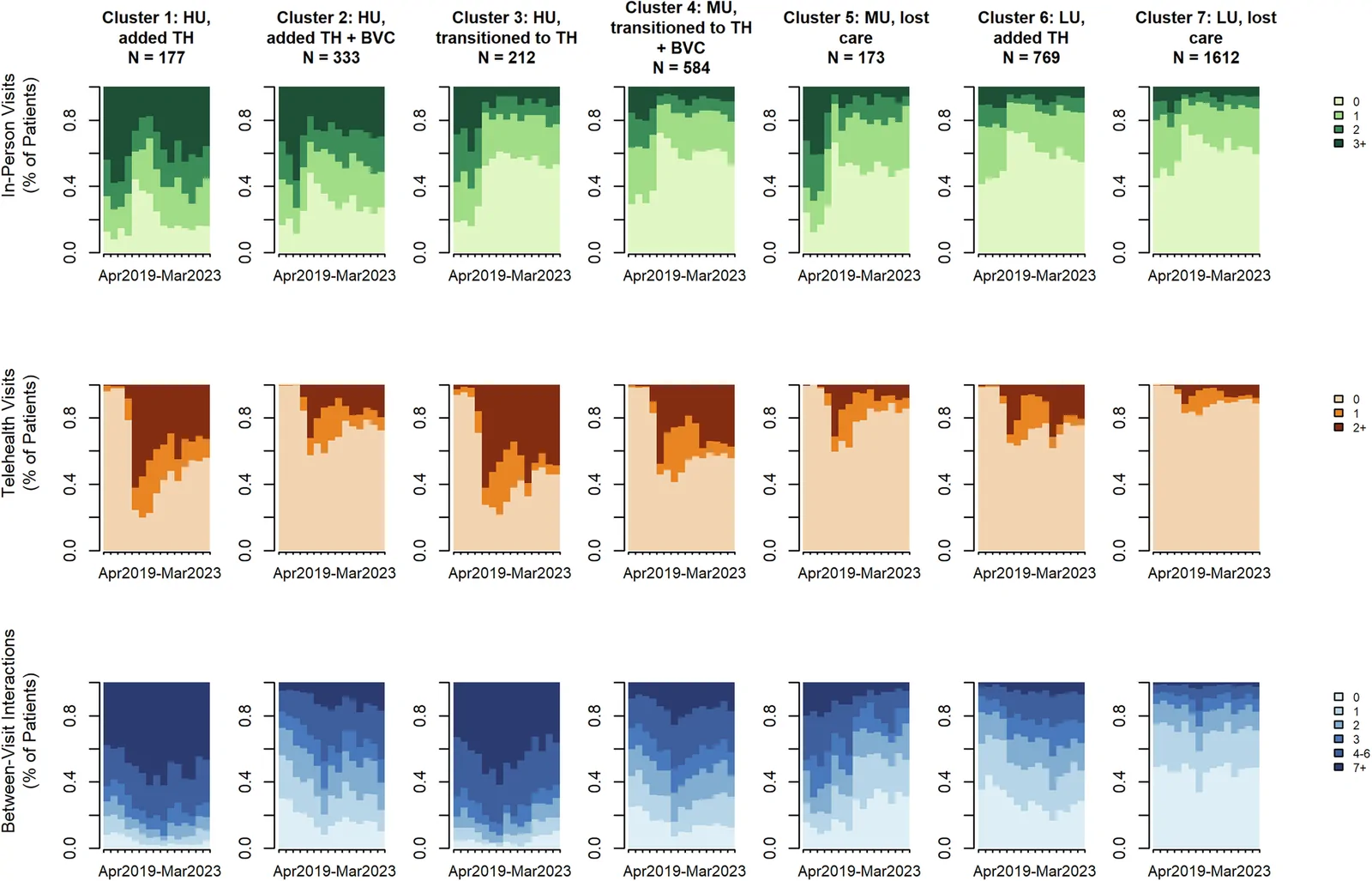
State distribution plots of clusters at UCSF by care modality. The state distribution plot shows the proportion of individuals in each care state (in-person, telehealth, and between-visit interactions) at each time point. The *x*-axis represents time (e.g., months or encounters), and the stacked colored areas indicate the relative frequency of each state. Changes in the height of each colored band over time reflect shifts in the overall composition of care states across the cohort, allowing visualization of dominant modalities and temporal trends in care engagement. HU high utilizer, MU moderate utilizer, LU low utilizer, TH telehealth, BVC between-visit care interactions.

We identified seven distinct care utilization clusters as the final number of clusters within the UCSF diabetes cohort, representing diverse patterns of engagement with in-person, telehealth, and between-visit care modalities over time. Figure 1 displays the distribution of each care modality at each time point by cluster. Cluster labels represent qualitative descriptors based on visual interpretation and were not derived from quantitatively specified definitions. Index plots showing all the individual trajectories in each cluster are displayed in Supplementary Fig. 1.

Cluster 1, *High Utilizers who added Telehealth* (*n* = 177), had very high in-person and between-visit care before the pandemic. About a year after the shelter-in-place mandate, they returned to very high in-person care and sustained high levels of between-visit care alongside moderate telehealth uptake. Cluster 2, *High utilizers who added Telehealth and Between-Visit care* (*n* = 333), had high in-person and moderate between-visit care before the pandemic. Following the shelter-in-place mandate, they returned to prior in-person care levels, sustained moderate increases in telehealth, and increased between-visit care. Cluster 3, *High utilizers who transitioned to Telehealth* (*n* = 212), had high in-person, very high between-visit care, and some telehealth use before the pandemic. Post-shelter-in-place, they shifted to high telehealth and between-visit care without returning to prior in-person rates. Cluster 4, *Moderate utilizers who transitioned to Telehealth and Between-Visit Care* (*n* = 584), had moderate in-person and between-visit care before the pandemic. Post-shelter-in-place, they shifted to moderate telehealth and increased between-visit care without returning to prior in-person rates. Cluster 5, *Moderate utilizers who lost care* (*n* = 173), had moderate in-person and between-visit care before the pandemic but did not recover either modality after the shelf-in-place period, and telehealth uptake was minimal. Cluster 6, *Low utilizers who added Telehealth* (*n* = 769), had low in-person and between-visit care before the pandemic. Post-shelter-in-place, they returned nearly to prior in-person rates, while adding telehealth and slightly increasing between-visit care. Cluster 7, *Low utilizers who lost care* (*n* = 1612), had low utilization across all modalities before and after the shelter-in-place mandate. They did not return to pre-pandemic in-person rates, demonstrated minimal telehealth uptake, and had no between-visit care.

Table 2 details the seven clusters classified by pre-pandemic utilization and post-pandemic changes in in-person, telehealth, and between-visit care trajectories over time. For comparisons of sociodemographic and clinical characteristics, clusters were grouped into three post-pandemic trajectories: added digital care (telehealth and/or between-visit care), transitioned to digital care (telehealth and/or between-visit care), and lost care. Among the 3860 patients, 33% added digital care, 21% transitioned to digital care, and 46% lost care. Sociodemographic composition varied significantly across trajectories (Table 3; full descriptive tables for each cluster are presented in Supplementary Table 1). Patients who added digital care were more likely to be female (*p* = 0.02) and Black/African American (*p* < 0.001), or Hispanic with non-English language preference (*p* < 0.001) compared to patients who lost care or transitioned to digital care. These patients were also slightly more likely to be covered by Medicaid (*p* = 0.031), have high comorbidity scores (CCI 3+) (*p* < 0.001), and uncontrolled A1c at baseline (*p* < 0.001). Patients who transitioned to digital care were more likely to be White, Black/African American, or Hispanic with an English-language preference (*p* < 0.001), and be covered by Medicaid or Medicare (*p* = 0.031) compared to patients in other care trajectories. Individuals who transitioned to digital care also had higher comorbidity scores (CCI 3+) (*p* < 0.001), were less likely to have missing A1c or blood pressure values (*p* < 0.001 and *p* = 0.001, respectively), and were more likely to have an active patient portal at baseline (*p* < 0.001). Patients who lost care were more likely to be male (*p* = 0.02), Asian—particularly with a non-English language preference (*p* < 0.001), and commercially insured (*p* = 0.031) compared to patients in other care trajectories. Patients who lost care were also more likely to have lower comorbidity scores (CCI 0–2) (*p* < 0.001), missing baseline A1c or blood pressure results (*p* < 0.001 and *p* = 0.001, respectively), and low patient portal enrollment (*p* < 0.001).

**Table 2:** Descriptions of UCSF healthcare trajectories by cluster

Pre-pandemic utilization	Cluster	*N* (%)	How each care pattern changed relative to pre-pandemic			Post-pandemic care trajectory
			In-person	Telehealth	Between visits	
High utilizers	UCSF 1	177 (4.6%)	Returned to pre-levels	High initial uptake, moderate sustained use	Continued high usage with a slight increase overall	Added telehealth
High utilizers	UCSF 2	333 (8.6%)	Returned to almost pre-levels	Sustained moderate uptake	Moderate uptake with a slight increase overall	Added telehealth and between-visit care
High utilizers	UCSF 3	212 (5.5%)	Did not recover to pre-levels	Sustained high uptake	Continued high usage (no sustained increase)	Transitioned to telehealth
Moderate utilizers	UCSF 4	584 (15.1%)	Did not recover to pre-levels	Sustained moderate uptake	Moderate uptake with a slight increase overall	Transitioned to telehealth and between-visit care
Moderate utilizers	UCSF 5	173 (4.5%)	Did not recover to pre-levels	Moderate uptake not sustained	Moderate usage, decreasing trends	Lost care
Low utilizers	UCSF 6	769 (19.9%)	Returned to almost pre-levels	Sustained moderate uptake	Low uptake with an increase overall	Added telehealth
Low utilizers	UCSF 7	1612 (41.8%)	Did not recover to pre-levels	Low, consistent non-usage	Low, consistent non-usage	Lost care

We defined adding digital care as in-person visit rates returning to pre-pandemic levels, while telehealth and/or between-visit care remain elevated. Those who transitioned to digital care saw in-person visit rates remain below pre-pandemic levels, while telehealth and between-visit care increased to replace in-person care. Those who lost care had minimal uptake of between-visit care or telehealth, even though in-person care rates did not rebound. We also further specified whether those who added or transitioned to digital care primarily adopted telehealth, between-visit care, or both.

**Table 3:** Sociodemographic characteristics of care trajectories at UCSF

	Total *N* (%)	Added digital care	Transitioned to digital care	Lost care	*P* value
		Clusters 1, 2, 6	Clusters 3, 4	Clusters 5, 7	
***N*** **(%)**	*N* = 3860	1279 (33)	796 (21)	1785 (46)	
Age category (%)					0.079
18–34	91 (2)	28 (2)	22 (3)	41 (2)	
35–49	472 (12)	155 (12)	113 (14)	204 (11)	
50–64	1234 (32)	419 (33)	267 (34)	548 (31)	
65–74	1118 (29)	347 (27)	227 (29)	544 (30)	
75+	945 (24)	330 (26)	167 (21)	448 (25)	
Sex (%)					0.020
Female	2091 (54)	726 (57)	440 (55)	925 (52)	
Male	1769 (46)	553 (43)	356 (45)	860 (48)	
Joint race ethnicity and language (%)					<0.001
Asian and English preference	1050 (27)	327 (26)	150 (19)	573 (32)	
Asian and non-English preference	478 (12)	145 (11)	55 (7)	278 (16)	
Black/African American	527 (14)	216 (17)	141 (18)	170 (10)	
Hispanic and English preference	366 (9)	109 (9)	86 (11)	171 (10)	
Hispanic and non-English preference	114 (3)	52 (4)	17 (2)	45 (3)	
Other/Un and English preference	317 (8)	104 (8)	66 (8)	147 (8)	
Other/Un and non-English preference	67 (2)	31 (2)	6 (1)	30 (2)	
White	941 (24)	295 (23)	275 (35)	371 (21)	
Race or ethnicity (%)					<0.001
Asian	1528 (40)	472 (37)	205 (26)	851 (48)	
Black/African American	527 (14)	216 (17)	141 (18)	170 (10)	
Hispanic/Latino	480 (12)	161 (13)	103 (13)	216 (12)	
Other/Unknown	384 (10)	135 (11)	72 (9)	177 (10)	
White	941 (24)	295 (23)	275 (35)	371 (21)	
Language preference (%)					<0.001
Chinese	296 (8)	93 (7)	25 (3)	178 (10)	
English	3154 (82)	1029 (80)	714 (90)	1411 (79)	
Other	298 (8)	105 (8)	42 (5)	151 (8)	
Spanish	112 (3)	52 (4)	15 (2)	45 (3)	
Insurance (%)					0.031
Commercial	1708 (44)	559 (44)	329 (41)	820 (46)	
Medicaid	502 (13)	187 (15)	120 (15)	195 (11)	
Medicare	1644 (43)	531 (42)	347 (44)	766 (43)	
Uninsured	1 (0)	0 (0)	0 (0)	1 (0)	
Missing	5 (0)	2 (0)	0 (0)	3 (0)	
Neighborhood SES (1 = lowest) (%)					0.056
1	651 (17)	221 (17)	157 (20)	273 (15)	
2	689 (18)	245 (19)	144 (18)	300 (17)	
3	752 (19)	232 (18)	147 (18)	373 (21)	
4	886 (23)	304 (24)	158 (20)	424 (24)	
5	698 (18)	219 (17)	149 (19)	330 (18)	
Missing	184 (5)	58 (5)	41 (5)	85 (5)	
Charlson score (%)					<0.001
0–2	2982 (77)	946 (74)	545 (68)	1491 (84)	
3+	878 (23)	333 (26)	251 (32)	294 (16)	
Baseline A1c control (%)					<0.001
Uncontrolled A1c (≥8)	818 (21)	289 (23)	192 (24)	337 (19)	
Controlled A1c (<8)	2611 (68)	849 (66)	549 (69)	1213 (68)	
Missing	431 (11)	141 (11)	55 (7)	235 (13)	
Baseline BP control (%)					0.001
BP ≤ 120/80	658 (17)	220 (17)	147 (18)	291 (16)	
BP ≤ 140/90	2126 (55)	716 (56)	430 (54)	980 (55)	
BP > 140/90	930 (24)	301 (24)	206 (26)	423 (24)	
Missing	146 (4)	42 (3)	13 (2)	91 (5)	
Patient portal enrollment (%)					<0.001
No	861 (22)	293 (23)	97 (12)	471 (26)	
Yes	2999 (78)	986 (77)	699 (88)	1314 (74)	
Missing					

*SES* socioeconomic status, *A1c* hemoglobin A1c lab result, *BP* blood pressure.

*P values compare patient characteristics by care trajectory based on the Chi-squared test for categorical measures.

### SFHN

After excluding patients without a visit in the last 12 months of the study period, we identified a cohort of 6811 patients with diabetes at SFHN (Table 1). The SFHN diabetes cohort was younger than the UCSF cohort, with two-thirds of patients under the age of 65. Most patients at this health system were non-English speakers (57%), including 28% identifying as Hispanic and Spanish preference and 26% identifying as Asian and Chinese preference. Most patients were insured through Medicaid (40%), Medicare (31%), or Healthy Workers (15%), and nearly two-thirds (64%) resided in neighborhoods within the lowest two nSES quintiles. While 85% of patients had lower comorbidity scores (CCI < 3), 39% had uncontrolled A1c at baseline. Digital engagement was notably low, with less than 10% of patients having an active MyChart account at baseline.

**Fig. 2:**
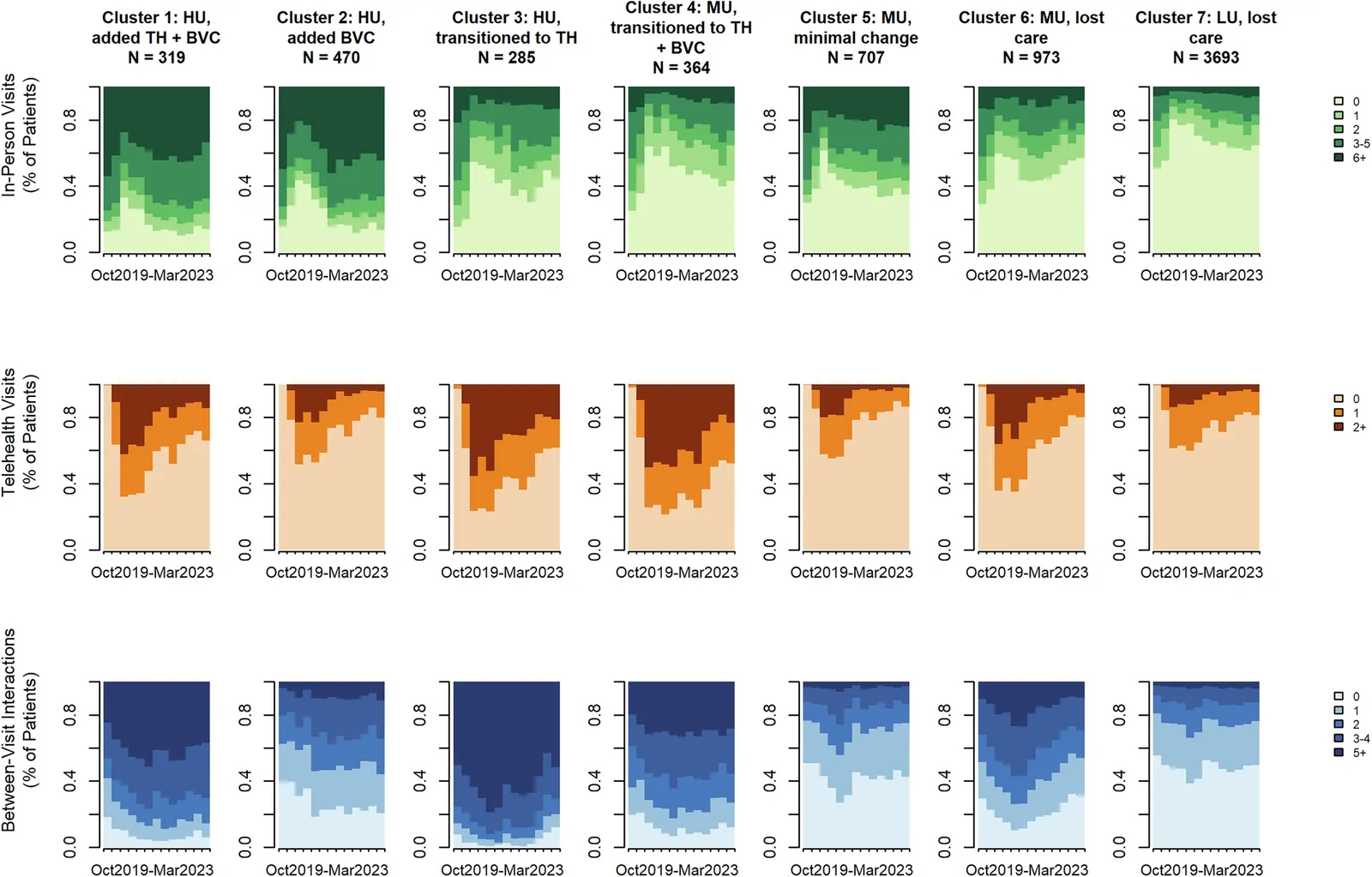
State distribution plots of clusters at SFHN by care modality. The state distribution plot shows the proportion of individuals in each care state (in-person, telehealth, and between-visit interactions) at each time point. The *x*-axis represents time (e.g., months or encounters), and the stacked colored areas indicate the relative frequency of each state. Changes in the height of each colored band over time reflect shifts in the overall composition of care states across the cohort, allowing visualization of dominant modalities and temporal trends in care engagement. HU high utilizer, MU moderate utilizer, LU low utilizer, TH telehealth, BVC between-visit care interactions.

We identified seven care utilization clusters as the final number of clusters within the SFHN cohort. Figure 2 displays the distribution of each care modality at each time point by cluster. Cluster labels represent qualitative descriptors based on visual interpretation and were not derived from quantitatively specified definitions. Index plots showing all the individual trajectories are displayed in Supplementary Fig. 2.

Cluster 1, *High utilizers who added Telehealth and Between-Visit care* (*n* = 319), had high in-person and between-visit care prior to the pandemic. Within 1 year of the shelter-in-place mandate, they returned to pre-pandemic in-person care levels while adding more between-visit care and moderate telehealth uptake. Cluster 2, *High utilizers who added Between-Visit care* (*n* = 470), had high in-person and moderate between-visit care before the pandemic. A year after the shelter-in-place mandate was lifted, they returned to prior in-person rates while increasing between-visit care. Telehealth uptake was minimal and not sustained over time. Cluster 3, *High utilizers who transitioned to Telehealth* (*n* = 285), had high in-person and between-visit care with some telehealth use before the pandemic. Post-shelter-in-place, they did not return to pre-pandemic in-person care levels but maintained high between-visit care and had high adoption of telehealth. Cluster 4, *Moderate utilizers who transitioned to Telehealth and Between-Visit care* (*n* = 364), had moderate in-person and high between-visit engagement prior to the pandemic. Post-shelter-in-place, they did not return to pre-pandemic in-person levels but had high telehealth uptake and increased between-visit care. Cluster 5, *Moderate utilizers with minimal change* (*n* = 707), had moderate in-person and low between-visit care prior to the pandemic. After the shelter-in-place mandate lifted, their in-person care levels nearly returned to pre-pandemic levels, with slight telehealth uptake and between-visit engagement that was not sustained over time. Cluster 6, *Moderate utilizers who lost care* (*n* = 973), had moderate in-person and between-visit care utilization prior to the pandemic. After the shelter-in-place mandate, they did not recover in-person care levels, which continued to decline over time, with any telehealth and between-visit care also trending downward. Cluster 7, *Low utilizers who lost care* (*n* = 3693), had low in-person and between-visit utilization before the pandemic. Post-shelter-in-place, they did not return to in-person levels and maintained minimal between-visit care, with any telehealth uptake not sustained.

We classified the seven SFHN clusters by pre-pandemic utilization and post-pandemic changes in in-person, telehealth, and between-visit care (Table 4) and grouped them into four trajectories: added digital care (telehealth and/or between-visit care), transitioned to digital care (telehealth and/or between-visit care), lost care, and sustained minimal change (Table 5, full descriptive tables for each cluster are presented in Supplementary Table 2). Patients who added digital care were more likely to be ≥50 years old (*p* = 0.037), Hispanic with a non-English language preference (*p* < 0.001), and insured through Medicaid or Medicare (*p* < 0.001) compared to other care trajectories. Clinically, these patients were also more likely to have high comorbidity scores (CCI 3+) (*p* < 0.001) and uncontrolled A1c levels at baseline (*p* < 0.001). Patients who transitioned to digital care were more likely to be 75+ years old (*p* = 0.037), Black/African American or White (*p* < 0.001), and much less likely to have a non-English language preference (*p* < 0.001) compared to other care trajectories. These patients were most likely to be covered by Medicare, had high comorbidity scores (CCI 3+), had controlled baseline A1c levels, and had an active patient portal at baseline (*p* < 0.001 for all characteristics). Patients who lost care were more likely to be Asian—particularly with a non-English language preference (*p* < 0.001) and be enrolled in Healthy Workers (*p* < 0.001), compared to other care trajectories. Clinically, these patients were more likely to have lower comorbidity scores (CCI 0–2), missing baseline A1c and blood pressure results, and low patient portal enrollment (*p* < 0.001 for all characteristics). SFHN patients who had minimal change in care patterns were more likely to be 35–49 years old (*p* = 0.037), Hispanic/Latino with a non-English language preference (*p* < 0.001), and uninsured (*p* < 0.001) compared to other care trajectories. Clinically, they were also more likely to have lower comorbidity scores (CCI 0–2), slightly more likely to have uncontrolled A1c at baseline, and a very low likelihood of being enrolled in the patient portal (*p* < 0.001 for all characteristics).

**Table 4:** Descriptions of SFHN healthcare trajectories by cluster

Pre-pandemic utilization	Cluster	*N* (%)	How each care pattern changed relative to pre-pandemic			Post-pandemic care trajectory
			In-person	Telehealth	Between visits	
High utilizers	SFHN 1	319 (4.7%)	Returned to pre-levels	Moderate initial uptake, some sustained use	Continued high usage with a slight increase overall	Added telehealth and between-visit care
High utilizers	SFHN 2	470 (6.9%)	Returned to pre-levels	Low uptake not sustained	Moderate uptake with a slight increase overall	Added between-visit care
High utilizers	SFHN 3	285 (4.2%)	Did not recover to pre-levels	High initial uptake, moderate sustained use	Continued high usage (no sustained increase)	Transitioned to telehealth
Moderate utilizers	SFHN 4	364 (5.3%)	Did not recover to pre-levels	High initial uptake, moderate sustained use	Continued high usage with a slight increase overall	Transitioned to telehealth and between-visit care
Moderate utilizers	SFHN 5	707 (10.4%)	Returned to pre-levels	Low uptake not sustained	Low uptake with an increase overall	Minimal change
Moderate utilizers	SFHN 6	973 (14.3%)	Did not recover to pre-levels	Moderate uptake not sustained	Moderate usage, decreasing trends	Lost care
Low utilizers	SFHN 7	3693 (54.2%)	Did not recover to pre-levels	Low uptake not sustained	Low, consistent non-usage	Lost care

We defined adding digital care as in-person visit rates returning to pre-pandemic levels, while telehealth and/or between-visit care remain elevated. Those who transitioned to digital care saw in-person visit rates remain below pre-pandemic levels, while telehealth and between-visit care increased to replace in-person care. Those who lost care had minimal uptake of between-visit care or telehealth, even though in-person care rates did not rebound. Those with minimal change experienced minimal changes to their care utilization patterns from before the pandemic. We also further specified whether those who added or transitioned to digital care primarily adopted telehealth, between-visit care, or both.

**Table 5:** Sociodemographic characteristics of care trajectories at SFHN

	Total	Added digital care	Transitioned to digital care	Lost care	Minimal change	*P* value*
		Clusters 1, 2	Clusters 3, 4	Clusters 6, 7	Cluster 5	
***N*** **(%)**	*N* = 6811	*N* = 789 (12)	*N* = 649 (10)	*N* = 4666 (69)	*N* = 707 (10)	
Age category (%)						0.037
18–34	162 (2)	10 (1)	16 (2)	123 (3)	13 (2)	
35–49	1080 (16)	118 (15)	101 (16)	723 (15)	138 (20)	
50–64	3215 (47)	378 (48)	308 (47)	2201 (47)	328 (46)	
65–74	1727 (25)	201 (25)	156 (24)	1214 (26)	156 (22)	
75+	627 (9)	82 (10)	68 (10)	405 (9)	72 (10)	
Sex (%)						0.094
Female	3581 (53)	394 (50)	372 (57)	2463 (53)	352 (50)	
Male	3229 (47)	395 (50)	277 (43)	2202 (47)	355 (50)	
Joint race ethnicity and language (%)						<0.001
Asian and English preference	641 (9)	62 (8)	62 (10)	446 (10)	71 (10)	
Asian and non-English preference	1753 (26)	94 (12)	85 (13)	1442 (31)	132 (19)	
Black/African American	1027 (15)	116 (15)	176 (27)	659 (14)	76 (11)	
Hispanic and English preference	449 (7)	57 (7)	64 (10)	276 (6)	52 (7)	
Hispanic and non-English preference	1881 (28)	340 (43)	103 (16)	1137 (24)	301 (43)	
Other/Un and English preference	254 (4)	24 (3)	33 (5)	173 (4)	24 (3)	
Other/Un and non-English preference	84 (1)	13 (2)	5 (1)	56 (1)	10 (1)	
White	722 (11)	83 (11)	121 (19)	477 (10)	41 (6)	
Race or ethnicity (%)						<0.001
Asian	2394 (35)	156 (20)	147 (23)	1888 (40)	203 (29)	
Black/African American	1027 (15)	116 (15)	176 (27)	659 (14)	76 (11)	
Hispanic/Latino	2330 (34)	397 (50)	167 (26)	1413 (30)	353 (50)	
Other/Unknown	338 (5)	37 (5)	38 (6)	229 (5)	34 (5)	
White	722 (11)	83 (11)	121 (19)	477 (10)	41 (6)	
Language preference (%)						<0.001
Chinese	1324 (19)	52 (7)	67 (10)	1130 (24)	75 (11)	
English	2931 (43)	335 (42)	437 (67)	1908 (41)	251 (36)	
Other	655 (10)	55 (7)	44 (7)	480 (10)	76 (11)	
Spanish	1901 (28)	347 (44)	101 (16)	1148 (25)	305 (43)	
Insurance (%)						<0.001
Commercial	71 (1)	6 (1)	6 (1)	53 (1)	6 (1)	
Healthy workers	1049 (15)	44 (6)	53 (8)	875 (19)	77 (11)	
Medicaid	2709 (40)	364 (46)	274 (42)	1806 (39)	265 (37)	
Medicare	2126 (31)	283 (36)	278 (43)	1359 (29)	206 (29)	
Uninsured	828 (12)	92 (12)	38 (6)	546 (12)	152 (21)	
Missing	28 (0)	0 (0)	0 (0)	27 (1)	1 (0)	
Neighborhood SES (1 = lowest) (%)						0.053
1	2561 (38)	306 (39)	261 (40)	1728 (37)	266 (38)	
2	1771 (26)	227 (29)	147 (23)	1199 (26)	198 (28)	
3	1049 (15)	122 (15)	88 (14)	737 (16)	102 (14)	
4	889 (13)	78 (10)	92 (14)	634 (14)	85 (12)	
5	405 (6)	40 (5)	48 (7)	280 (6)	37 (5)	
Missing	136 (2)	16 (2)	13 (2)	88 (2)	19 (3)	
Charlson score (%)						<0.001
0–2	5837 (86)	576 (73)	481 (74)	4162 (89)	618 (87)	
3+	974 (14)	213 (27)	168 (26)	504 (11)	89 (13)	
Baseline A1c control (%)						<0.001
Uncontrolled A1c (≥8)	2631 (39)	376 (48)	272 (42)	1677 (36)	306 (43)	
Controlled A1c (<8)	3307 (49)	364 (46)	337 (52)	2273 (49)	333 (47)	
Missing	873 (13)	49 (6)	40 (6)	716 (15)	68 (10)	
Baseline BP control (%)						<0.001
BP ≤ 120/80	1629 (24)	198 (25)	143 (22)	1134 (24)	154 (22)	
BP ≤ 140/90	3598 (53)	427 (54)	351 (54)	2418 (52)	402 (57)	
BP > 140/90	1205 (18)	147 (19)	149 (23)	783 (17)	126 (18)	
Missing	379 (6)	17 (2)	6 (1)	331 (7)	25 (4)	
Patient portal enrollment (%)						<0.001
No	6217 (91)	724 (92)	524 (81)	4293 (92)	676 (96)	
Yes	594 (9)	65 (8)	125 (19)	373 (8)	31 (4)	

*SES* socioeconomic status, *A1c* hemoglobin A1c lab result, *BP* blood pressure.

*P values compare patient characteristics by care trajectory based on the Chi-squared test for categorical measures.

### Key patterns in care trajectories across health systems

Next, we descriptively compared these trajectories across healthcare systems to identify important patterns and/or differences. UCSF saw higher proportions of patients who added or transitioned to digital care, with 33% of patients adding digital care and 21% transitioning to digital care (Table 3). In contrast, SFHN had lower proportions in these categories: 12% added digital care and 10% transitioned to digital care (Table 5). At UCSF, those who added digital care (Clusters UCSF 1, UCSF 2, UCSF 6) demonstrated sustained moderate to high telehealth uptake and varying rates of between-visit interactions, but with an increase in use overall (Fig. 1). At SFHN, however, those who added digital care (Clusters SFHN 1, SFHN 2) showed low to moderate uptake of telehealth (which was not sustained) and higher volumes of between-visit care (Fig. 2).

Across both health systems, those who transitioned to digital care (Clusters UCSF 3, UCSF 4, SFHN 3, SFHN 4) showed moderate to high uptake of telehealth. Patients who transitioned to digital care had heterogeneous uptake of between-visit care, as some clusters increased their interactions (Clusters UCSF 4, SFHN 4) while others maintained moderate to high uptake (with no sustained increase in volume; Clusters UCSF 3, SFHN 3).

SFHN exhibited higher proportions of patients who either maintained prior care patterns with minimal changes or experienced care disengagement: 10% maintained in-person visit rates with minimal to no uptake of digital care, and 69% experienced care disruptions and lost care (compared to 46% of patients who lost care at UCSF). Patients who lost care (Clusters UCSF 5, UCSF 7, SFHN 6, SFHN 7) demonstrated low adoption of telehealth and between-visit care, often characterized by consistent non-usage or decreasing trends in use. In-person visit rates also did not recover to pre-pandemic levels among the clusters that lost care.

We also examined how trajectories differed by pre-pandemic utilization levels across both systems (see Supplementary Note 1 for a detailed description). High utilizers mostly returned to their pre-pandemic in-person rates of care and incorporated both telehealth and/or between-visit care with sustained use of between-visit care modalities. Moderate utilizers had more heterogeneity, with some transitioning to digital care, some losing care, and some showing minimal changes. Low utilizers were the largest cluster in both health systems; some low utilizers lost care, while some added telehealth care.

Sensitivity analyses using optimal matching (OM) instead of dynamic hamming distance (DHD) produced broadly similar trajectory types. While OM yielded some variation in the distribution of patients, the overall care patterns and substantive interpretations remained consistent with the DHD results (Supplementary Fig. 3).

## Discussion

In a large, diverse sample of patients with T2DM across two large San Francisco health systems, we characterized clusters of multi-modal healthcare utilization patterns across three types of visits (in-person, telehealth visits, and between-visit encounters) from April 2019 to March 2023 using sequence and cluster analysis. Across both UCSF and SFHN, three distinct patterns emerged: adding digital care, where in-person visit volumes returned to baseline while telehealth and between-visit communications remained elevated; transitioning to digital care, characterized by sustained reductions in in-person visits alongside increased telehealth and/or between-visit use; and loss of care, with neither in-person nor digital services returning to pre-pandemic levels. At SFHN, a fourth pattern—minimal change—was observed, reflecting stable use of in-person visits with persistently low use of telehealth and between-visit care. Within these patterns, we noticed heterogeneity not only in the degree of telehealth uptake and sustainability of between-visit care, but also in the timing of these shifts—differences that were captured through our use of sequence analysis—highlighting nuanced variation both across and within health systems. We then examined whether sociodemographic and clinical factors were associated with distinct healthcare utilization trajectories. Our findings provide critical insights into the factors shaping transitions to digital care, sustained in-person use, and care loss.

A key finding was the identification of characteristics among the clusters that adopted digital care. Several clusters reflected a transition from primarily in-person care to primarily digital engagement after the shelter-in-place mandate, which was more common among Black or White patients with an English-language preference, those covered by Medicare, and those with higher comorbidity burdens. These patterns suggest that digital modalities may be particularly attractive or accessible for managing complex health needs or for those facing barriers to consistent in-person care, such as transportation, work schedules, or mobility issues, which are more pronounced among marginalized groups^44,45^. These results align with the existing literature, which indicates the value of hybrid models of care in increasing accessibility and supporting chronic disease management^4,6,7,9,29,44–46^. These findings are particularly insightful because they both corroborate prior literature on digital shifts in care delivery and extend it to a broader population, including both academic and safety-net settings. By analyzing two health systems serving demographically distinct populations, we demonstrate that digital care adoption is not limited to well-resourced populations but also emerges as a critical tool for sustaining care engagement in more diverse, underserved settings. Notably, clusters at UCSF, which relied more heavily on video visits, exhibited greater telehealth uptake than clusters at SFHN (where telehealth was predominantly phone visits)^30^. This pattern is somewhat unexpected, as phone-based care is believed to be broadly accessible to populations from diverse socioeconomic backgrounds; therefore, in SFHN, where phone-based visits were the predominant form of telehealth visits, we expected greater uptake of telehealth^30,47^.

Race and ethnicity, preferred language, and health insurance were associated with each care trajectory, yet their distributions differed across systems. This heterogeneity implies that demographic factors do not determine care utilization but can vary depending on the context, such as the health system’s capacity or digital health outreach approach. Notably, patterns by race and ethnicity and preferred language were similar across both health systems. Patients who added digital care were more likely to be Spanish-speaking Hispanic, particularly at SFHN; patients who transitioned to digital care were more likely to be White or Black, and those who lost care were more likely to be Asian with a non-English language preference. Differences by race and ethnicity and language indicate that cultural and linguistic factors may shape how patients engage with digital care, reflecting persistent barriers as well as opportunities to enhance access. System-level elements, such as language-concordant communication, digital literacy support, and targeted outreach, likely play a critical role in determining which patients are able to adopt and sustain digital care use, underscoring the need for equity-oriented implementation strategies^23,48,49^. The higher uptake of digital care observed among Spanish-speaking individuals may have multiple explanations. This pattern could reflect successful system-level efforts to offer and support digital care adoption for Spanish speakers. It may also reflect differences in care-seeking patterns or structural barriers to in-person care. Spanish-speaking patients may face greater time, transportation, or logistical constraints, which could increase the relative value and utilization of remote care modalities^50^.

Moreover, there was notable heterogeneity in health insurance coverage across the two health systems, highlighting how structural context may shape care engagement. These differences reflect the broader roles of each system: UCSF as a tertiary referral center with a commercially insured base, and SFHN as a safety-net system serving populations with more limited financial and digital access. Insurance status corresponded closely to care trajectories. At UCSF, patients who lost care were more likely to be commercially insured, a group that may experience weaker continuity with a single health system due to broader care alternatives, potentially reflecting data loss rather than true change in care patterns. At SFHN, uninsured patients were overrepresented among those who lost care or exhibited minimal changes in utilization. For uninsured patients, financial barriers and limited access options likely constrain sustained engagement, while for commercially insured patients, greater flexibility in provider choice may diffuse continuity across systems. Insurance type functions as a structural determinant of healthcare engagement, influencing not only access but also the stability and modality of care over time. Health systems serving distinct payer mixes, therefore, face different equity challenges: safety-net systems must continue to address affordability and digital access, while commercially oriented systems may need to strengthen coordination and continuity within fragmented care networks. Addressing these gaps through targeted interventions—such as insurance navigation support, proactive outreach, and integrated digital care supports—may improve continuity and mitigate disparities across diverse insurance contexts. Additionally, examining how policy-level factors, such as insurance or network restrictions, influence care engagement could inform system-wide strategies to prevent care loss across diverse populations.

When examining care trajectories through the lens of patients’ pre-pandemic utilization levels, clear trends emerged across both health systems. High utilizers prior to the pandemic were the most likely to adopt or transition to digital care, demonstrating moderate to high telehealth uptake and between-visit care engagement. This suggests that the most engaged patients were more likely to incorporate digital modalities into their care. Notably, there was one UCSF cluster of low utilizers that also added care, demonstrating some heterogeneity. No clusters among moderate utilizers showed consistent trends of adding care.

Those who transitioned to digital care tended to be moderate to high utilizers prior to the pandemic, adopting telehealth and between-visit care as a partial substitute for lost in-person visits. However, for both the trajectories that added digital care or transitioned to digital care, sustained between-visit growth was not universal, suggesting that engagement in care between visits may ebb and flow depending on patients’ needs and interests. Future studies should expand upon this work to evaluate if and how between-visit care supports care access.

Patients who lost care constituted the largest segment at both systems, mainly comprised of low or moderate-pre-pandemic utilizers with limited telehealth uptake. This pattern underscores how individuals with historically lower engagement may be particularly vulnerable to care disruption and highlights the limited capacity of digital modalities alone to re-engage these patients. Notably, SFHN had a larger group of low utilizers who added care, suggesting that leveraging more accessible modalities of engagement (such as greater emphasis on telephone calls versus video visits) may mitigate disengagement.

These results underscore the complex interplay between digital health adoption, socioeconomic factors, and healthcare disparities in urban contexts. By examining how patients navigate and combine multiple care modalities, we can better understand how these options may create complexity, overwhelm, or a mismatch with patient needs, ultimately leading to reduced engagement.

In this study, Chinese-speaking patients were disproportionately represented among those who lost care, whereas Spanish-speaking patients were frequently observed among trajectories characterized by the addition of digital care, despite also being represented among those with minimal utilization changes. This nuanced pattern differs from prior studies, which have often reported that both Chinese and Spanish speakers face barriers to digital tool adoption and are more likely to struggle with specific tasks required to use digital health tools effectively, but it still illustrates that linguistic and cultural barriers influence how patients access and interact with different modalities and aspects of care^18,51,52^. The relatively higher prevalence of digital care uptake among Spanish-speaking patients in our study may reflect unmeasured differences in age (which we did not adjust for as our aim was to compare demographics across clusters), as well as contextual factors not captured in our data, including differences in care needs, perceived value of telehealth, targeted system-level outreach, or unmeasured structural barriers to in-person care. Prior work at SFHN suggests that language-concordant digital skills training particularly benefits Spanish speakers and patients with lower digital engagement, raising the possibility that organizational strategies may influence utilization patterns^53^. Future research should more directly examine the mechanisms underlying language-specific differences in digital care adoption, including the roles of patient preferences, structural constraints, and health system interventions to address barriers and reduce disparities in engagement among those at risk of care disengagement.

The differences in cluster patterns at UCSF and SFHN also indicate that digital engagement is not one-size-fits-all, and the importance of tailoring digital adoption strategies to the structural and patient-level realities of each organization, including digital preferences and needs^30,44,45^. Clusters at SFHN demonstrated moderate to low telehealth engagement, compared to UCSF, which had multiple clusters with high telehealth adoption; but SFHN patients were more likely to have sustained or increased their between-visit engagement. This highlights the continued importance of telephone calls (as opposed to patient portal messages) as an approach to engage historically excluded populations^30^.

Lastly, future studies should consider integrating clinical outcomes by using regression models to examine associations between utilization care trajectory clusters and longitudinal health outcomes, such as glycemic control, blood pressure, or hospitalizations. Longitudinal regression analyses will further clarify whether digital care adoption translates into measurable health benefits, and for whom.

This study has some limitations. First, as this is an observational study using electronic health records (EHR) data, we could not measure relevant factors such as patient motivation, access to digital devices and internet, lifestyle factors, social drivers, or social norms that may influence access to or attitude towards diabetes and remote engagement in care. While we included a range of clinical and sociodemographic covariates, selection into different care modalities is non-random and likely reflects underlying health engagement, care-seeking behavior, or attitudes toward different care modalities. Because our covariates were measured at baseline, we were unable to take into consideration how the progression of comorbidities and new health conditions throughout the study period might influence utilization over time^54^.

Inclusion in the study was restricted to those who had at least one interaction with the healthcare system both pre- and post-pandemic, and results are therefore only representative of an empaneled, care-seeking population. Relatedly, we were not able to include information on visits that participants may have received outside of UCSF or SFHN. Because clusters were derived independently within each system and the healthcare systems differed in telehealth implementation and patient sociodemographic composition, cross-system comparisons are descriptive and may not reflect directly comparable utilization trajectories. Lastly, results are likely not generalizable to rural settings due to differences in digital access and infrastructure.

In two urban health system cohorts of adults with Type 2 diabetes analyzed over multiple years, we found that care utilization trajectories differed by sociodemographic and clinical characteristics. Specifically, in the post-pandemic era, we saw a substantial proportion of the patient population at both health systems embrace digital care (both telehealth and between-visit care), suggesting that increased digital accessibility supplements and allows patients to engage in care in ways that better suit their needs. However, a significant proportion of patients either lost access to care and experienced care disruptions or did not adopt digital care, suggesting a need for future research and tailored strategies to ensure equity in digital engagement and to close gaps in care continuity. As healthcare systems continue to expand hybrid models of care, it is critical to ensure equitable access and effectiveness across diverse populations. Future work should build upon this research and explore ways to mitigate disparities in the use of all care modalities, particularly for diverse patients and healthcare systems.

## Methods

### Study setting and data source

We conducted a 4-year retrospective longitudinal cohort study of adults with T2DM receiving care at two health systems in San Francisco: the University of California, San Francisco (UCSF) and the San Francisco Health Network (SFHN). UCSF is an academic tertiary care center with four primary care practices. SFHN is an integrated, urban safety-net health system with 14 primary care practices. The analytic study period was April 1, 2019 to March 31, 2023 at UCSF and October 1, 2019 to March 31, 2023 at SFHN. The later SFHN start date for utilization analyses was due to the adoption of a new EHR software in August 2019 at SFHN.

We used EHR from the two health systems, capturing sociodemographic data, clinical characteristics, and encounter data. Since the study procedures involved only a review of data collected for routine clinical care, informed consent was waived due to minimal risk to patients in the cohort. All study procedures were approved by the UCSF Institutional Review Board (IRB #20-31253) in accordance with the Declaration of Helsinki. We report our procedures and results in accordance with the STROBE guidelines.

### Study sample

We identified a cohort of adult patients ≥18 years old with T2DM who were assigned a primary care provider as of April 1, 2019, at UCSF and SFHN. The creation of the cohort is described in detail in a previous study^30^. In brief, patients with T2DM were identified based on the following criteria before the study start date: an active DM health maintenance modifier; having a T2DM-related ICD-9/ICD-10 code on their active problem list; the most recent HbA1c test result ≥6.5%, and/or having an active diabetes medication on their medication list. To ensure active engagement in care, patients were only included if they had at least one encounter with their diabetes (primary care or endocrinology) team between April 1, 2019 to March 31, 2021, and at least one healthcare interaction (including patient portal messages, phone calls) in the last 12 months of the study period (April 1, 2022 to March 31, 2023). Although the SFHN study period for longitudinal utilization analyses began on October 1, 2019, cohort eligibility for both health systems was defined as of April 1, 2019. Pre-October 2019 SFHN EHR data were used only to ascertain cohort inclusion criteria and were not used in the utilization sequence analysis.

### Exposure variables

We included a comprehensive set of patient-level sociodemographic characteristics as of 4/1/2019 that were extracted from the EHR. We included age (as a continuous variable and categorized as 18–34, 35–49, 50–64, 65–74, or 75+), race and ethnicity (categorized as Hispanic/Latino(a); non-Hispanic Asian; non-Hispanic Black; non-Hispanic White/Caucasian; or other non-Hispanic races, which included American Indian or Alaskan Native, Native Hawaiian or Other Pacific Islander), preferred language (English, Chinese, Spanish, or Other), sex (male/female), health insurance as of April 2019 (Commercial/Private, Medicare, Medicaid, Healthy Workers [an SF-specific program that provides health insurance coverage to people working in In-Home Supportive Services, foster care, or other publicly funded roles who do not qualify for traditional Medicaid or employer-sponsored coverage], or Uninsured), and neighborhood socioeconomic status (nSES) based on geocoded addresses, and categorized as quintiles from low to high^55^.

Clinical characteristics included the Charlson comorbidity index (CCI, modified to exclude diabetes) from 04/01/2019-03/31/2020, baseline A1c as of 04/01/2019, and baseline blood pressure control as of 04/01/2019^56,57^. Baseline A1c control was categorized as: controlled A1c (A1c ≤8), uncontrolled A1c (A1c >8), or missing. Baseline blood pressure was categorized as ≤120/80 (no hypertension), ≤140/90 (controlled hypertension), or >140/90 mmHg (uncontrolled hypertension). Baseline patient portal enrollment status was included as a proxy measure of digital access at the beginning of the study period^58^. It was extracted from the EHR as of 4/1/2020 and documented as enrolled vs. not enrolled at the time of study inclusion.

### Construction of care trajectories and cluster analysis

We conducted sequence and cluster analysis to group patients into homogeneous clusters of outpatient primary care/diabetes-related healthcare utilization over time, based on the number of in-person, telehealth, and between-visit interactions per quarter across the study period. This approach enabled us to measure the similarity of each patient’s sequence for each encounter modality across quarters and cluster similar sequences together^37^. For example, a patient whose sequence includes frequent in-person visits early in the study period followed by a shift to telehealth visits may be grouped in a cluster representing patients who transitioned from traditional to digital care modalities. This distinction is particularly important in settings where patients engage with multiple modalities of care, as trajectory analysis may overlook interdependencies and transitions between the modalities^59–61^. To account for multiple modalities (in-person visits, telehealth visits, and between-visit interactions), we employed multichannel (also called multidimensional) sequence analysis, which allows creating three distinct channels, one for each encounter type^62^. We conducted analyses separately for each health system to ensure clusters were generated based on care receipt rather than system-level factors, as prior studies have shown health system differences are significant^54,63^. Analyses were conducted in R Version 4.2.1 using the “TraMineR,” “cluster,” and “UniversalCVI” packages^64–67^.

First, for each health system, we summarized care utilization across three distinct types of patient encounters with the healthcare team (comprised of physicians, advanced practice providers, nurses, social workers, pharmacists, dietitians, medical assistants, or embedded behavioral health clinicians), which represented the three channels of the multichannel sequence analysis: *in-person visits*, defined as scheduled in-person visits with a member of the care team*; telehealth visits*, defined as scheduled video or telephone visits with a member of the care team*;* and *between-visit interactions*, defined as unscheduled telephone calls or patient portal messages occurring between visits.

In both health systems, Q2 2020 (April–June 2020) was excluded from the analysis due to the unique encounter patterns during the COVID-19 Shelter-in-Place period, during which utilization habits varied widely, and EHR utilization accuracy was much lower because standard workflows, operating procedures, and documentation protocols took several months to develop. To address this in our analysis, we removed that quarter from the dataset, effectively creating a skip over Q2 2020 and treating Q1 2020 and Q3 2020 as consecutive quarters. We examined the distribution of the number of quarterly encounters by each type and created binned variables for each encounter type. Bins were selected by choosing the number of encounters in the 90th percentile as the highest and ensuring the sample size in each bin was sufficient. The final bins, referred to as “states” in sequence analysis, were then applied to each modality. Specifically, for in-person visits, quarterly counts were binned as 0, 1, 2, 3+ at UCSF, and 0, 1, 2, 3–5, 6+ at SFHN. For telehealth visits, quarterly counts were binned as 0, 1, 2+ at UCSF, and 0, 1, 2 at SFHN. For between-visit interactions, quarterly counts were binned as 0, 1, 2, 3, 4–6, 7+ at UCSF, and 0, 1, 2, 3–4, 5+ at SFHN. For each patient, the binned quarterly encounter counts for each modality were then used to construct individual care utilization sequences.

After building the individual sequences, we quantified differences between sequences for each channel using the Dynamic Hamming Distance (DHD) method^68^. This approach quantifies the similarity between sequences by calculating the "cost" required to transform one sequence into another, based on substitutions of states to make sequences identical. The DHD approach calculates time-varying substitution costs based on transitions between states such that states with more frequent transitions between consecutive time points are assigned lower costs. Compared to alternative approaches to calculate sequence similarity, DHD is more sensitive to the timing of care patterns, as opposed to duration and order of states^38,69^. Because we were particularly interested in the timing of events (i.e., when patients’ healthcare utilization increases or decreases), we chose DHD over alternative dissimilarity measures. Calculating dissimilarity between each pair of sequences resulted in a dissimilarity matrix for each channel, comparing each sequence to all other sequences in the data. The three dissimilarity matrices were then averaged to obtain a final, multichannel dissimilarity matrix.

Lastly, we grouped patients into healthcare utilization clusters using agglomerative hierarchical clustering with Ward’s linkage. To determine the optimal number of clusters, we evaluated three cluster quality measures—Hubert’s C index (HC), the average silhouette width (ASW), and the Calinski–Harabasz (CH) index to identify cluster solutions with high homogeneity within clusters and distinction between clusters (Supplementary Fig. 4). Together, these metrics provided complementary evidence to identify the most stable and interpretable cluster solutions, ranging from *k* = 2 to *k* = 10. Since several cluster solutions may have similar quality, we also used expert judgment to make considerations on parsimony and salience, and ultimately chose a slightly higher number of clusters to capture heterogeneities in healthcare utilization^70^.

### Interpretation of cluster patterns

To interpret differences in care trajectories across clusters, we conducted qualitative visual comparisons of cluster-specific utilization patterns. Specifically, we examined state distribution plots of quarterly encounter frequencies by modality and cluster to identify overarching patterns in care trajectories over time. These visual comparisons allowed us to characterize clusters according to salient trends in utilization, such as increases, decreases, or shifts between in-person, telehealth, and between-visit interactions. Visual interpretation was used to contextualize and describe cluster patterns after cluster solutions were identified.

To ease our comparison of clusters within and between health systems, we conducted post-hoc analyses to categorize clusters based on similar overall trends in utilization. We grouped clusters into categories based on their trajectories after the shelter-in-place mandate was lifted, relative to their pre-pandemic behavior. We further characterized clusters by pre-pandemic in-person utilization levels.

### Statistical analyses

We described patient characteristics overall and by healthcare utilization trajectory. In addition to the exposure variables listed above, we included a joint variable for race, ethnicity, and preferred language to capture the intersectional and integrated nature of these characteristics. Categorical variables were compared using chi-squared tests. Analyses were conducted separately for each health system, with cross-system comparisons of care trajectories and pre-pandemic utilization presented descriptively. To characterize average care utilization by cluster, we descriptively plotted mean encounters per patient, per quarter, by modality within each cluster (Supplementary Fig. 5).

### Sensitivity analyses

Sensitivity analyses were conducted to assess the robustness of the clustering approach, ensuring the stability of cluster solutions under different assumptions and methodologies. As an alternative to Dynamic Hamming, our primary approach, we calculated the dissimilarity between trajectories using the Optimal Matching (OM) algorithm, the dissimilarity measure that has received the most attention in sequence analysis^37^. OM has been shown to prioritize duration over timing and order of events^69^. After repeating the sequence and cluster analysis using OM, we compared the resulting cluster solutions and interpretations to test the stability of our clusters under different assumptions and analytic decisions.

## Supplementary information

Supplementary Information

## Data Availability

The datasets generated and analyzed during the current study cannot be shared publicly due to the privacy of individuals who participated in the study and institutional data use agreements governing electronic health record data from UCSF and SFHN. De-identified data may be shared upon reasonable request to the corresponding author, subject to institutional review board approval and applicable data governance requirements.

## Code availability

R Version 4.2.1 was used to perform data analysis. In particular, we used the “TraMineR,” “cluster,” and “UniversalCVI” packages to conduct analyses. The code for sequence analysis may be shared upon request to the corresponding author.

## References

[1] Rodriguez, J. A., Saadi, A., Schwamm, L. H., Bates, D. W. & Samal, L. Disparities in telehealth use among California patients with limited English proficiency. *Health Aff.* **40**, 487–495 (2021). 10.1377/hlthaff.2020.00823 33646862

[2] Ramsetty, A. & Adams, C. Impact of the digital divide in the age of COVID-19. *J. Am. Med. Inform. Assoc.* **27**, 1147–1148 (2020). 10.1093/jamia/ocaa078 PMC7197532 32343813

[3] Robinson, J., Borgo, L., Fennell, K. & Funahashi, T. T. The Covid-19 pandemic accelerates the transition to virtual care. *NEJM Catal*. 10.1056/CAT.20.0399 (2020).

[4] Reed, M. et al. Outpatient electronic health records and the clinical care and outcomes of patients with diabetes mellitus. *Ann. Intern. Med.* **157**, 482–489 (2012). 10.7326/0003-4819-157-7-201210020-00004 PMC3603566 23027319

[5] Herrin, J. et al. The effectiveness of implementing an electronic health record on diabetes care and outcomes. *Health Serv. Res.* **47**, 1522–1540 (2012). 10.1111/j.1475-6773.2011.01370.x PMC3401397 22250953

[6] Harris, L. T., Koepsell, T. D., Haneuse, S. J., Martin, D. P. & Ralston, J. D. Glycemic control associated with secure patient-provider messaging within a shared electronic medical record. *Diabetes Care* **36**, 2726–2733 (2013). 10.2337/dc12-2003 PMC3747898 23628618

[7] Lyles, C., Schillinger, D. & Sarkar, U. Connecting the dots: health information technology expansion and health disparities. *PLOS Med.* **12**, e1001852 (2015). 10.1371/journal.pmed.1001852 PMC4501812 26172977

[8] Kruse, C. S., Argueta, D. A., Lopez, L. & Nair, A. Patient and provider attitudes toward the use of patient portals for the management of chronic disease: a systematic review. *J. Med. Internet Res.* **17**, e40 (2015). 10.2196/jmir.3703 PMC4376181 25707035

[9] Goldzweig, C. L. et al. Electronic patient portals: evidence on health outcomes, satisfaction, efficiency, and attitudes: a systematic review. *Ann. Intern. Med.* **159**, 677–687 (2013). 10.7326/0003-4819-159-10-201311190-00006 24247673

[10] Rodriguez, J. A., Casillas, A., Cook, B. L. & Marlin, R. P. The language of equity in digital health: prioritizing the needs of limited English proficient communities in the Patient Portal 2.0. *J. Health Care Poor Underserved* **32**, 211–219 (2021).

[11] Ralston, J. D. et al. Patient use of secure electronic messaging within a shared medical record: a cross-sectional study. *J. Gen. Intern. Med.* **24**, 349–355 (2009). 10.1007/s11606-008-0899-z PMC2642567 19137379

[12] Lyles, C. R. et al. Using electronic health record portals to improve patient engagement: research priorities and best practices. *Ann. Intern. Med.* **172**, S123–S129 (2020). 10.7326/M19-0876 PMC7800164 32479176

[13] Antonio, M. G., Petrovskaya, O. & Lau, F. Is research on patient portals attuned to health equity? A scoping review. *J. Am. Med. Inform. Assoc.* **26**, 871–883 (2019). 10.1093/jamia/ocz054 PMC7647227 31066893

[14] Sarkar, U. et al. Social disparities in internet patient portal use in diabetes: evidence that the digital divide extends beyond access. *J. Am. Med. Inform. Assoc.* **18**, 318–321 (2011). 10.1136/jamia.2010.006015 PMC3078675 21262921

[15] Deshpande, N., Arora, V. M., Vollbrecht, H., Meltzer, D. O. & Press, V. eHealth literacy and patient portal use and attitudes: cross-sectional observational study. *JMIR Hum. Factors* **10**, e40105 (2023). 10.2196/40105 PMC9919456 36705947

[16] Ancker, J. S. et al. Use of an electronic patient portal among disadvantaged populations. *J. Gen. Intern. Med.* **26**, 1117–1123 (2011). 10.1007/s11606-011-1749-y PMC3181304 21647748

[17] Ancker, J. S., Hafeez, B. & Kaushal, R. Socioeconomic disparities in adoption of personal health records over time. *Am. J. Manag. Care* **22**, 539–540 (2016). PMC5474311 27541700

[18] Khoong, E. C., Rivadeneira, N. A., Hiatt, R. A. & Sarkar, U. The use of technology for communicating with clinicians or seeking health information in a multilingual urban cohort: cross-sectional survey. *J. Med. Internet Res.* **22**, e16951 (2020). 10.2196/16951 PMC7171563 32250280

[19] Tieu, L. et al. Online patient websites for electronic health record access among vulnerable populations: portals to nowhere? *J. Am. Med. Inform. Assoc.* **24**, e47–e54 (2017). 10.1093/jamia/ocw098 PMC6080722 27402138

[20] Gibbons, M. C. A historical overview of health disparities and the potential of eHealth solutions. *J. Med. Internet Res.* **7**, e50 (2005). 10.2196/jmir.7.5.e50 PMC1550690 16403714

[21] Eberly, L. A. et al. Patient characteristics associated with telemedicine access for primary and specialty ambulatory care during the COVID-19 pandemic. *JAMA Netw. Open* **3**, e2031640 (2020). 10.1001/jamanetworkopen.2020.31640 PMC7772717 33372974

[22] Ryskina, K. L., Shultz, K., Zhou, Y., Lautenbach, G. & Brown, R. T. Older adults’ access to primary care: gender, racial, and ethnic disparities in telemedicine. *J. Am. Geriatr. Soc.* **69**, 2732–2740 (2021). 10.1111/jgs.17354 PMC8497419 34224577

[23] Sarkar, U. et al. The literacy divide: health literacy and the use of an internet-based patient portal in an integrated health system—results from the Diabetes Study of Northern California (DISTANCE). *J. Health Commun.* **15**, 183–196 (2010). 10.1080/10810730.2010.499988 PMC3014858 20845203

[24] Davies, A. R., Honeyman, M. & Gann, B. Addressing the digital inverse care law in the time of COVID-19: potential for digital technology to exacerbate or mitigate health inequalities. *J. Med. Internet Res.* **23**, e21726 (2021). 10.2196/21726 PMC8030655 33735096

[25] Lyles, C. R. et al. Patient race/ethnicity and shared medical record use among diabetes patients. *Med. Care* **50**, 434 (2012). 10.1097/MLR.0b013e318249d81b 22354209

[26] Anthony, D. L., Campos-Castillo, C. & Lim, P. S. Who isn’t using patient portals and why? Evidence and implications from a national sample of US adults. *Health Aff.* **37**, 1948–1954 (2018). 10.1377/hlthaff.2018.05117 30633673

[27] Lyles, C. R., Nguyen, O. K., Khoong, E. C., Aguilera, A. & Sarkar, U. Multilevel determinants of digital health equity: a literature synthesis to advance the field. *Annu. Rev. Public Health* **44**, 383–405 (2023). 10.1146/annurev-publhealth-071521-023913 PMC10329412 36525960

[28] Alkureishi, M. A. et al. Digitally disconnected: qualitative study of patient perspectives on the digital divide and potential solutions. *JMIR Hum. Factors* **8**, e33364 (2021). 10.2196/33364 PMC8675564 34705664

[29] Nouri, S., Khoong, E. C., Lyles, C. R. & Karliner, L. Addressing equity in telemedicine for chronic disease management during the Covid-19 pandemic. *NEJM Catal.* **1**, (2020).

[30] Nouri, S. et al. Visit and between-visit interaction frequency before and after COVID-19 telehealth implementation. *JAMA Netw. Open* **6**, e2333944 (2023). 10.1001/jamanetworkopen.2023.33944 PMC10504619 37713198

[31] Li, P. et al. Changes in racial and ethnic disparities in glucose-lowering drug utilization and glycated haemoglobin A1c in US adults with diabetes: 2005-2018. *Diabetes Obes. Metab.* **25**, 516–525 (2023). 10.1111/dom.14894 PMC9812896 36251173

[32] Sheon, A. R., Bolen, S. D., Callahan, B., Shick, S. & Perzynski, A. T. Addressing disparities in diabetes management through novel approaches to encourage technology adoption and use. *JMIR Diabetes* **2**, e16 (2017). 10.2196/diabetes.6751 PMC6238861 30291090

[33] Lyles, C. R., Grothaus, L., Reid, R. J., Sarkar, U. & Ralston, J. D. Communication about diabetes risk factors during between-visit encounters. *Am. J. Manag. Care* **18**, 807–815 (2012). 23286610

[34] Rodriguez, J. A., Betancourt, J. R., Sequist, T. D., & Ganguli, I. Differences in the use of telephone and video telemedicine visits during the COVID-19 pandemic. *Am. J. Manag. Care* **27**, 21–26 (2021). 10.37765/ajmc.2021.88573 PMC10877492 33471458

[35] Nath, B. et al. Trends in electronic health record inbox messaging during the COVID-19 pandemic in an ambulatory practice network in New England. *JAMA Netw. Open* **4**, e2131490 (2021). 10.1001/jamanetworkopen.2021.31490 PMC8511977 34636917

[36] Patel, S. Y. et al. Trends in outpatient care delivery and telemedicine during the COVID-19 pandemic in the US. *JAMA Intern. Med.* **181**, 388–391 (2021). 10.1001/jamainternmed.2020.5928 PMC7670397 33196765

[37] Abbott, A. & Tsay, A. Sequence analysis and optimal matching methods in sociology. *Sociol. Methods Res.* **29**, 3–33 (2020).

[38] Emery, K. & Berchtold, A. Comparison of two approaches in multichannel sequence analysis using the Swiss Household Panel. *Longitud. Life Course Stud.* **14**, 592–623 (2023). 10.1332/175795921X16698302233894 37874200

[39] Eisenberg-Guyot, J. et al. Life-course trajectories of employment quality and health in the U.S.: a multichannel sequence analysis. *Soc. Sci. Med.* **264**, 113327 (2020). 10.1016/j.socscimed.2020.113327 PMC7607590 32919256

[40] Savaré, L., Ieva, F., Corrao, G. & Lora, A. Capturing the variety of clinical pathways in patients with schizophrenic disorders through state sequences analysis. *BMC Med. Res. Methodol.* **23**, 174 (2023). 10.1186/s12874-023-01993-7 PMC10386768 37516839

[41] Kempa-Liehr, A. W. et al. Healthcare pathway discovery and probabilistic machine learning. *Int. J. Med. Inf.* **137**, 104087 (2020). 10.1016/j.ijmedinf.2020.104087 32126509

[42] Mathew, S., Peat, G., Parry, E., Sokhal, B. S. & Yu, D. Applying sequence analysis to uncover ‘real-world’ clinical pathways from routinely collected data: a systematic review. *J. Clin. Epidemiol.* **166**, 111226 (2024). 10.1016/j.jclinepi.2023.111226 38036188

[43] Cezard, G., Sullivan, F. & Keenan, K. Understanding multimorbidity trajectories in Scotland using sequence analysis. *Sci. Rep.* **12**, 16485 (2022). 10.1038/s41598-022-20546-4 PMC9526700 36182953

[44] Institute of Medicine (US) Committee on Quality of Health Care in America. *Crossing the Quality Chasm: A New Health System for the 21st Century* (National Academies Press (US), 2001). 25057539

[45] Choxi, H., VanDerSchaaf, H., Li, Y. & Morgan, E. Telehealth and the Digital divide: identifying potential care gaps in video visit use. *J. Med. Syst.* **46**, 58 (2022). 10.1007/s10916-022-01843-x PMC9361960 35906432

[46] Coughlin, S. S., Williams, L. B. & Hatzigeorgiou, C. A systematic review of studies of web portals for patients with diabetes mellitus. *mHealth* **3**, 23–23 (2017). 10.21037/mhealth.2017.05.05 PMC5505929 28736732

[47] Uscher-Pines, L. et al. Changes in in-person, audio-only, and video visits In California’s Federally Qualified Health Centers, 2019–2022. *JAMA* **329**, 1219 (2023). 10.1001/jama.2023.1307 PMC10091174 37039799

[48] Khoong, E. C. et al. Patient interest in and barriers to telemedicine video visits in a multilingual urban safety-net system. *J. Am. Med. Inform. Assoc.* **28**, 349–353 (2021). 10.1093/jamia/ocaa234 PMC7883976 33164063

[49] Lee, G. et al. Understanding and addressing the digital health literacy needs of low-income limited English proficient Asian American patients. *Health Equity* **6**, 494–499 (2022). 10.1089/heq.2022.0045 PMC9518790 36186613

[50] Grossman, L. V. et al. Interventions to increase patient portal use in vulnerable populations: a systematic review. *J. Am. Med. Inform. Assoc.* **26**, 855–870 (2019). 10.1093/jamia/ocz023 PMC6696508 30958532

[51] Tieu, L. et al. A self-report measure of digital skills needed to use digital health tools among older adults—the Skills Measurement and Readiness Training for Digital Health (SMART Digital Health) Scale. *J. Am. Med. Inform. Assoc.* 10.1093/jamia/ocaf151 (2025). PMC12626212 40971609

[52] Rapson, T. et al. Identifying skill and usability barriers to digital health tool use among patients in safety net clinics: a mixed methods study. *JMIR Hum. Factors* **13**, e78430 (2026). 10.2196/78430 PMC13138792 42081796

[53] Rapson, T. et al. Impact of digital support interventions on patient portal use among patients in an urban safety-net health system. (2016).

[54] Pacca, L. et al. Using sequence and cluster analysis to characterize variables that unfold over time: implementation and practical considerations for epidemiologists. *Am. J. Epidemiol.* kwaf065. 10.1093/aje/kwaf065 (2025). PMC12952161 40219634

[55] Gomez, S. L. et al. The California Neighborhoods Data System: a new resource for examining the impact of neighborhood characteristics on cancer incidence and outcomes in populations. *Cancer Causes Control* **22**, 631–647 (2011). 10.1007/s10552-011-9736-5 PMC3102646 21318584

[56] Pritchard, E. et al. Combining Charlson and Elixhauser scores with varying lookback predicated mortality better than using individual scores. *J. Clin. Epidemiol.* **130**, 32–41 (2021). 10.1016/j.jclinepi.2020.09.020 33002637

[57] Quan, H. et al. Coding algorithms for defining comorbidities in ICD-9-CM and ICD-10 administrative data. *Med. Care* **43**, 1130–1139 (2005). 10.1097/01.mlr.0000182534.19832.83 16224307

[58] Perzynski, A. T. et al. Patient portals and broadband internet inequality. *J. Am. Med. Inform. Assoc.* **24**, 927–932 (2017). 10.1093/jamia/ocx020 PMC6259664 28371853

[59] Agborsangaya, C. B., Lau, D., Lahtinen, M., Cooke, T. & Johnson, J. A. Health-related quality of life and healthcare utilization in multimorbidity: results of a cross-sectional survey. *Qual. Life Res.* **22**, 791–799 (2013). 10.1007/s11136-012-0214-7 22684529

[60] Segal, J. B., Sen, A. P., Glanzberg-Krainin, E. & Hutfless, S. Factors associated with overuse of health care within US health systems: a cross-sectional analysis of medicare beneficiaries from 2016 to 2018. *JAMA Health Forum* **3**, e214543 (2022). 10.1001/jamahealthforum.2021.4543 PMC8903118 35977230

[61] Cheng, Y., Goodin, A. J., Pahor, M., Manini, T. & Brown, J. D. Healthcare utilization and physical functioning in older adults in the United States. *J. Am. Geriatr. Soc.* **68**, 266–271 (2020). 10.1111/jgs.16260 31755551

[62] Gauthier, J.-A., Widmer, E. D., Bucher, P. & Notredame, C. Multichannel sequence analysis applied to social science data. *Sociol. Methodol.* **40**, 1–38 (2010).

[63] Shih, J. J. et al. Differences in telemedicine use for patients with diabetes in an academic versus safety net health system: retrospective cohort study. *J. Med. Internet Res.* **27**, e64635 (2025). 10.2196/64635 PMC11976178 40126552

[64] Gabadinho, A., Ritschard, G., Müller, N. S. & Studer, M. Analyzing and visualizing state sequences in R with TraMineR. *J. Stat. Softw.* **40**, 1–37 (2011).

[65] Maechler, M., Rousseeuw, P., Hubert, M. & Hornik, K. *cluster: Cluster Analysis Basics and Extensions*. (R package version 2.1.1, 2021).

[66] Wiroonsri, N. & Preedasawakul, O. A correlation-based fuzzy cluster validity index with secondary options detector. *Fuzzy Sets Syst.* **523**, 109632 (2024).

[67] Wiroonsri, N. Clustering performance analysis using a new correlation-based cluster validity index. *Pattern Recognit.* **145**, 109910 (2024).

[68] Lesnard, L. Using optimal matching analysis in sociology: cost setting and sociology of time. in *Advances in Sequence Analysis: Theory, Method, Applications* (eds Blanchard, P., Bühlmann, F. & Gauthier, J.-A.) 39–50. 10.1007/978-3-319-04969-4 (Springer, 2014).

[69] Studer, M. & Ritschard, G. What matters in differences between life trajectories: a comparative review of sequence dissimilarity measures. *J. R. Stat. Soc. Ser. A Stat. Soc.* **179**, 481–511 (2016).

[70] Liao, T. F. et al. Sequence analysis: its past, present, and future. *Soc. Sci. Res.* **107**, 102772 (2022). 10.1016/j.ssresearch.2022.102772 36058612

