# Supplementary material for: Identifying trajectories across care modalities before and after COVID-19 using sequence analysis
Source: NPJ Digit Med. 2026 May 26;9:759. doi: 10.1038/s41746-026-02766-4 (PMC13639023; doi:10.1038/s41746-026-02766-4)
Supplement: Supplementary file 1 [file 41746_2026_2766_MOESM1_ESM.docx]

**Supplementary Figure 1. Index Plots of Clusters at UCSF**

Index plots display the individual sequences for all the individuals in each cluster. Each horizontal line represents an individual sequence of care states over time, with colors denoting different modalities or states. The x-axis shows time (e.g., months or encounters), and the y-axis lists individuals, typically grouped by cluster. Blocks of the same color indicate stable periods within a care modality, while transitions between colors reflect shifts in care engagement or modality use. Patterns within and between clusters illustrate the overall heterogeneity and temporal structure of care trajectories.

**Supplementary Table 1. Descriptive Results of Clusters at UCSF**

|  | Total | 1: HU, added TH | 2: HU, added TH + BVC | 3: HU, transitioned to TH | 4: MU, transitioned to TH + BVC | 5: MU, lost care | 6: LU, added TH | 7: LU, lost care | Test | P-value |
| --- | --- | --- | --- | --- | --- | --- | --- | --- | --- | --- |
|  | N=3,860 | N=177 | N=333 | N=212 | N=584 | N=173 | N=769 | N=1,612 |  |  |
| Age (years) | 66 (56-74) | 67 (58-78) | 66 (56-75) | 62 (52-70) | 65 (54-73) | 67 (58-74) | 66 (54-74) | 66 (56-75) | Kruskal-Wallis | <0.001 |
| Age Category |  |  |  |  |  |  |  |  | Chi-square | 0.004 |
| 18-34 | 91 (2%) | 1 (0%) | 4 (1%) | 7 (3%) | 15 (3%) | 4 (2%) | 23 (3%) | 37 (2%) |  |  |
| 35-49 | 472 (12%) | 12 (7%) | 35 (11%) | 29 (14%) | 84 (14%) | 19 (11%) | 108 (14%) | 185 (11%) |  |  |
| 50-64 | 1,234 (32%) | 64 (36%) | 121 (36%) | 85 (40%) | 182 (31%) | 44 (25%) | 234 (30%) | 504 (31%) |  |  |
| 65-74 | 1,118 (29%) | 43 (24%) | 85 (26%) | 49 (23%) | 178 (30%) | 63 (36%) | 219 (28%) | 481 (30%) |  |  |
| 75+ | 945 (24%) | 57 (32%) | 88 (26%) | 42 (20%) | 125 (21%) | 43 (25%) | 185 (24%) | 405 (25%) |  |  |
| Sex |  |  |  |  |  |  |  |  | Chi-square | 0.002 |
| Female | 2,091 (54%) | 114 (64%) | 197 (59%) | 125 (59%) | 315 (54%) | 103 (60%) | 415 (54%) | 822 (51%) |  |  |
| Male | 1,769 (46%) | 63 (36%) | 136 (41%) | 87 (41%) | 269 (46%) | 70 (40%) | 354 (46%) | 790 (49%) |  |  |
| Joint Race Eth & Language |  |  |  |  |  |  |  |  | Chi-square | <0.001 |
| Asian x Pref Eng | 1,050 (27%) | 26 (15%) | 79 (24%) | 40 (19%) | 110 (19%) | 34 (20%) | 222 (29%) | 539 (33%) |  |  |
| Asian x Pref non-Eng | 478 (12%) | 17 (10%) | 49 (15%) | 10 (5%) | 45 (8%) | 30 (17%) | 79 (10%) | 248 (15%) |  |  |
| Black/AA | 527 (14%) | 51 (29%) | 72 (22%) | 43 (20%) | 98 (17%) | 29 (17%) | 93 (12%) | 141 (9%) |  |  |
| Hispanic x Pref Eng | 366 (9%) | 16 (9%) | 26 (8%) | 26 (12%) | 60 (10%) | 20 (12%) | 67 (9%) | 151 (9%) |  |  |
| Hispanic x Pref non-Eng | 114 (3%) |  | 14 (4%) | 3 (1%) | 14 (2%) | 10 (6%) | 25 (3%) | 35 (2%) |  |  |
| Other/Un x Pref Eng | 317 (8%) | 14 (8%) | 26 (8%) | 19 (9%) | 47 (8%) | 9 (5%) | 64 (8%) | 138 (9%) |  |  |
| Other/Un x Pref non-Eng | 67 (2%) | 2 (1%) | 9 (3%) | 4 (2%) | 2 (0%) | 1 (0%) | 20 (3%) | 29 (2%) |  |  |
| White | 941 (24%) | 38 (21%) | 58 (17%) | 67 (32%) | 208 (36%) | 40 (23%) | 199 (26%) | 331 (21%) |  |  |
| Race or Ethnicity |  |  |  |  |  |  |  |  | Chi-square | <0.001 |
| Asian | 1,528 (40%) | 43 (24%) | 128 (38%) | 50 (24%) | 155 (27%) | 64 (37%) | 301 (39%) | 787 (49%) |  |  |
| Black/AA | 527 (14%) | 51 (29%) | 72 (22%) | 43 (20%) | 98 (17%) | 29 (17%) | 93 (12%) | 141 (9%) |  |  |
| Hispanic/Latino | 480 (12%) | 29 (16%) | 40 (12%) | 29 (14%) | 74 (13%) | 30 (17%) | 92 (12%) | 186 (12%) |  |  |
| Other/Unknown | 384 (10%) | 16 (9%) | 35 (11%) | 23 (11%) | 49 (8%) | 10 (6%) | 84 (11%) | 167 (10%) |  |  |
| White | 941 (24%) | 38 (21%) | 58 (17%) | 67 (32%) | 208 (36%) | 40 (23%) | 199 (26%) | 331 (21%) |  |  |
| Language Preference |  |  |  |  |  |  |  |  | Chi-square | <0.001 |
| Chinese | 296 (8%) | 13 (7%) | 23 (7%) | 4 (2%) | 21 (4%) | 18 (10%) | 57 (7%) | 160 (10%) |  |  |
| English | 3,154 (82%) | 144 (81%) | 257 (77%) | 195 (92%) | 519 (89%) | 129 (75%) | 628 (82%) | 1,282 (80%) |  |  |
| Other | 298 (8%) | 7 (4%) | 40 (12%) | 10 (5%) | 32 (5%) | 16 (9%) | 58 (8%) | 135 (8%) |  |  |
| Spanish | 112 (3%) | 13 (7%) | 13 (4%) | 3 (1%) | 12 (2%) | 10 (6%) | 26 (3%) | 35 (2%) |  |  |
| Insurance |  |  |  |  |  |  |  |  | Chi-square | <0.001 |
| Commercial | 1,708 (44%) | 47 (27%) | 130 (39%) | 70 (33%) | 259 (44%) | 65 (38%) | 382 (50%) | 755 (47%) |  |  |
| Medicaid | 502 (13%) | 30 (17%) | 70 (21%) | 42 (20%) | 78 (13%) | 21 (12%) | 87 (11%) | 174 (11%) |  |  |
| Medicare | 1,644 (43%) | 100 (56%) | 132 (40%) | 100 (47%) | 247 (42%) | 86 (50%) | 299 (39%) | 680 (42%) |  |  |
| Uninsured | 1 (0%) | 0 (0%) | 0 (0%) | 0 (0%) | 0 (0%) | 0 (0%) | 0 (0%) | 1 (0%) |  |  |
| Missing | 5 (0%) | 0 (0%) | 1 (0%) | 0 (0%) | 0 (0%) | 1 (0%) | 1 (0%) | 2 (0%) |  |  |
| Neighborhood SES (1=lowest) |  |  |  |  |  |  |  |  | Chi-square | 0.003 |
| 1 | 651 (17%) | 37 (21%) | 69 (21%) | 54 (25%) | 103 (18%) | 33 (19%) | 115 (15%) | 240 (15%) |  |  |
| 2 | 689 (18%) | 37 (21%) | 79 (24%) | 41 (19%) | 103 (18%) | 33 (19%) | 129 (17%) | 267 (17%) |  |  |
| 3 | 752 (19%) | 32 (18%) | 53 (16%) | 38 (18%) | 109 (19%) | 35 (20%) | 147 (19%) | 338 (21%) |  |  |
| 4 | 886 (23%) | 36 (20%) | 78 (23%) | 33 (16%) | 125 (21%) | 38 (22%) | 190 (25%) | 386 (24%) |  |  |
| 5 | 698 (18%) | 30 (17%) | 40 (12%) | 37 (17%) | 112 (19%) | 25 (14%) | 149 (19%) | 305 (19%) |  |  |
| Missing | 184 (5%) | 5 (3%) | 14 (4%) | 9 (4%) | 32 (5%) | 9 (5%) | 39 (5%) | 76 (5%) |  |  |
| Charlson Score |  |  |  |  |  |  |  |  | Chi-square | <0.001 |
| 0-2 | 2,982 (77%) | 91 (51%) | 224 (67%) | 122 (58%) | 423 (72%) | 120 (69%) | 631 (82%) | 1,371 (85%) |  |  |
| 3+ | 878 (23%) | 86 (49%) | 109 (33%) | 90 (42%) | 161 (28%) | 53 (31%) | 138 (18%) | 241 (15%) |  |  |
| Baseline A1c Control |  |  |  |  |  |  |  |  | Chi-square | <0.001 |
| A1c >8 | 818 (21%) | 59 (33%) | 85 (26%) | 55 (26%) | 137 (23%) | 47 (27%) | 145 (19%) | 290 (18%) |  |  |
| A1c <8 | 2,611 (68%) | 109 (62%) | 222 (67%) | 151 (71%) | 398 (68%) | 124 (72%) | 518 (67%) | 1,089 (68%) |  |  |
| Missing | 431 (11%) | 9 (5%) | 26 (8%) | 6 (3%) | 49 (8%) | 2 (1%) | 106 (14%) | 233 (14%) |  |  |
| Baseline BP Control |  |  |  |  |  |  |  |  | Chi-square | <0.001 |
| BP <=120/80 | 658 (17%) | 33 (19%) | 57 (17%) | 47 (22%) | 100 (17%) | 38 (22%) | 130 (17%) | 253 (16%) |  |  |
| BP <=140/90 | 2,126 (55%) | 98 (55%) | 192 (58%) | 111 (52%) | 319 (55%) | 87 (50%) | 426 (55%) | 893 (55%) |  |  |
| BP >140/90 | 930 (24%) | 44 (25%) | 83 (25%) | 54 (25%) | 152 (26%) | 48 (28%) | 174 (23%) | 375 (23%) |  |  |
| Missing | 146 (4%) | 2 (1%) | 1 (0%) | 0 (0%) | 13 (2%) | 0 (0%) | 39 (5%) | 91 (6%) |  |  |
| Active MyChart |  |  |  |  |  |  |  |  | Chi-square | <0.001 |
| No | 861 (22%) | 50 (28%) | 108 (32%) | 17 (8%) | 80 (14%) | 52 (30%) | 135 (18%) | 419 (26%) |  |  |
| Yes | 2,999 (78%) | 127 (72%) | 225 (68%) | 195 (92%) | 504 (86%) | 121 (70%) | 634 (82%) | 1,193 (74%) |  |  |

Eth: Ethnicity, Pref: Preference, Eng: English, Non-Eng: Non-English, AA: African American, Un: Unknown, SES: socioeconomic status, A1c: hemoglobin A1c lab result, BP: blood pressure

**Supplementary Figure 2. Index Plots of Clusters at SFHN**

Index plots display the individual sequences for all the individuals in each cluster. Each horizontal line represents an individual sequence of care states over time, with colors denoting different modalities or states. The x-axis shows time (e.g., months or encounters), and the y-axis lists individuals, typically grouped by cluster. Blocks of the same color indicate stable periods within a care modality, while transitions between colors reflect shifts in care engagement or modality use. Patterns within and between clusters illustrate the overall heterogeneity and temporal structure of care trajectories.

**Supplementary Table 2. Descriptive Results of Clusters at SFHN**

|  | Total | 1: HU, added TH + BVC | 2: HU, added BVC | 3: HU, transitioned to TH | 4: MU, transitioned to TH + BVC | 5: MU, minimal change | 6: MU, lost care | 7: LU, lost care | Test | p-value |
| --- | --- | --- | --- | --- | --- | --- | --- | --- | --- | --- |
|  | N=6,811 | N=319 | N=470 | N=285 | N=364 | N=707 | N=973 | N=3,693 |  |  |
| Age (years) | 61 (53-67) | 61 (55-69) | 60 (53-67) | 61 (54-67) | 60 (52-68) | 60 (51-67) | 62 (54-69) | 60 (53-67) | Kruskal-Wallis | 0.001 |
| Age Category |  |  |  |  |  |  |  |  | Chi-square | 0.008 |
| 18-34 | 162 (2%) | 5 (2%) | 5 (1%) | 5 (2%) | 11 (3%) | 13 (2%) | 19 (2%) | 104 (3%) |  |  |
| 35-49 | 1,080 (16%) | 40 (13%) | 78 (17%) | 38 (13%) | 63 (17%) | 138 (20%) | 146 (15%) | 577 (16%) |  |  |
| 50-64 | 3,215 (47%) | 153 (48%) | 225 (48%) | 147 (52%) | 161 (44%) | 328 (46%) | 426 (44%) | 1,775 (48%) |  |  |
| 65-74 | 1,727 (25%) | 84 (26%) | 117 (25%) | 69 (24%) | 87 (24%) | 156 (22%) | 282 (29%) | 932 (25%) |  |  |
| 75+ | 627 (9%) | 37 (12%) | 45 (10%) | 26 (9%) | 42 (12%) | 72 (10%) | 100 (10%) | 305 (8%) |  |  |
| Sex |  |  |  |  |  |  |  |  | Chi-square | 0.005 |
| Female | 3,581 (53%) | 167 (52%) | 227 (48%) | 154 (54%) | 218 (60%) | 352 (50%) | 554 (57%) | 1,909 (52%) |  |  |
| Male | 3,229 (47%) | 152 (48%) | 243 (52%) | 131 (46%) | 146 (40%) | 355 (50%) | 418 (43%) | 1,784 (48%) |  |  |
| Missing | 1 (0%) | 0 (0%) | 0 (0%) | 0 (0%) | 0 (0%) | 0 (0%) | 1 (0%) | 0 (0%) |  |  |
| Joint Race Eth & Language |  |  |  |  |  |  |  |  | Chi-square | <0.001 |
| Asian x Pref Eng | 641 (9%) | 19 (6%) | 43 (9%) | 20 (7%) | 42 (12%) | 71 (10%) | 86 (9%) | 360 (10%) |  |  |
| Asian x Pref non-Eng | 1,753 (26%) | 43 (13%) | 51 (11%) | 21 (7%) | 64 (18%) | 132 (19%) | 191 (20%) | 1,251 (34%) |  |  |
| Black/AA | 1,027 (15%) | 56 (18%) | 60 (13%) | 97 (34%) | 79 (22%) | 76 (11%) | 219 (23%) | 440 (12%) |  |  |
| Hispanic x Pref Eng | 449 (7%) | 27 (8%) | 30 (6%) | 31 (11%) | 33 (9%) | 52 (7%) | 78 (8%) | 198 (5%) |  |  |
| Hispanic x Pref non-Eng | 1,881 (28%) | 117 (37%) | 223 (47%) | 41 (14%) | 62 (17%) | 301 (43%) | 210 (22%) | 927 (25%) |  |  |
| Other/Un x Pref Eng | 254 (4%) | 14 (4%) | 10 (2%) | 19 (7%) | 14 (4%) | 24 (3%) | 45 (5%) | 128 (3%) |  |  |
| Other/Un x Pref non-Eng | 84 (1%) | 6 (2%) | 7 (1%) | 1 (0%) | 4 (1%) | 10 (1%) | 13 (1%) | 43 (1%) |  |  |
| White | 722 (11%) | 37 (12%) | 46 (10%) | 55 (19%) | 66 (18%) | 41 (6%) | 131 (13%) | 346 (9%) |  |  |
| Race or Ethnicity |  |  |  |  |  |  |  |  | Chi-square | <0.001 |
| Asian | 2,394 (35%) | 62 (19%) | 94 (20%) | 41 (14%) | 106 (29%) | 203 (29%) | 277 (28%) | 1,611 (44%) |  |  |
| Black/AA | 1,027 (15%) | 56 (18%) | 60 (13%) | 97 (34%) | 79 (22%) | 76 (11%) | 219 (23%) | 440 (12%) |  |  |
| Hispanic/Latino | 2,330 (34%) | 144 (45%) | 253 (54%) | 72 (25%) | 95 (26%) | 353 (50%) | 288 (30%) | 1,125 (30%) |  |  |
| Other/Unknown | 338 (5%) | 20 (6%) | 17 (4%) | 20 (7%) | 18 (5%) | 34 (5%) | 58 (6%) | 171 (5%) |  |  |
| White | 722 (11%) | 37 (12%) | 46 (10%) | 55 (19%) | 66 (18%) | 41 (6%) | 131 (13%) | 346 (9%) |  |  |
| Language Preference |  |  |  |  |  |  |  |  | Chi-square | <0.001 |
| Chinese | 1,324 (19%) | 26 (8%) | 26 (6%) | 12 (4%) | 55 (15%) | 75 (11%) | 127 (13%) | 1,003 (27%) |  |  |
| English | 2,931 (43%) | 152 (48%) | 183 (39%) | 215 (75%) | 222 (61%) | 251 (36%) | 527 (54%) | 1,381 (37%) |  |  |
| Other | 655 (10%) | 21 (7%) | 34 (7%) | 17 (6%) | 27 (7%) | 76 (11%) | 110 (11%) | 370 (10%) |  |  |
| Spanish | 1,901 (28%) | 120 (38%) | 227 (48%) | 41 (14%) | 60 (16%) | 305 (43%) | 209 (21%) | 939 (25%) |  |  |
| Insurance |  |  |  |  |  |  |  |  | Chi-square | <0.001 |
| Commercial | 71 (1%) | 1 (0%) | 5 (1%) | 3 (1%) | 3 (1%) | 6 (1%) | 11 (1%) | 42 (1%) |  |  |
| Healthy Workers | 1,049 (15%) | 12 (4%) | 32 (7%) | 12 (4%) | 41 (11%) | 77 (11%) | 109 (11%) | 766 (21%) |  |  |
| Medicaid | 2,709 (40%) | 151 (47%) | 213 (45%) | 124 (44%) | 150 (41%) | 265 (37%) | 406 (42%) | 1,400 (38%) |  |  |
| Medicare | 2,126 (31%) | 139 (44%) | 144 (31%) | 136 (48%) | 142 (39%) | 206 (29%) | 362 (37%) | 997 (27%) |  |  |
| Uninsured | 828 (12%) | 16 (5%) | 76 (16%) | 10 (4%) | 28 (8%) | 152 (21%) | 82 (8%) | 464 (13%) |  |  |
| Missing | 28 (0%) | 0 (0%) | 0 (0%) | 0 (0%) | 0 (0%) | 1 (0%) | 3 (0%) | 24 (1%) |  |  |
| Neighborhood SES (1=lowest) |  |  |  |  |  |  |  |  | Chi-square | 0.020 |
| 1 | 2,561 (38%) | 136 (43%) | 170 (36%) | 129 (45%) | 132 (36%) | 266 (38%) | 356 (37%) | 1,372 (37%) |  |  |
| 2 | 1,771 (26%) | 83 (26%) | 144 (31%) | 54 (19%) | 93 (26%) | 198 (28%) | 267 (27%) | 932 (25%) |  |  |
| 3 | 1,049 (15%) | 52 (16%) | 70 (15%) | 34 (12%) | 54 (15%) | 102 (14%) | 143 (15%) | 594 (16%) |  |  |
| 4 | 889 (13%) | 28 (9%) | 50 (11%) | 34 (12%) | 58 (16%) | 85 (12%) | 125 (13%) | 509 (14%) |  |  |
| 5 | 405 (6%) | 16 (5%) | 24 (5%) | 26 (9%) | 22 (6%) | 37 (5%) | 65 (7%) | 215 (6%) |  |  |
| Missing | 136 (2%) | 4 (1%) | 12 (3%) | 8 (3%) | 5 (1%) | 19 (3%) | 17 (2%) | 71 (2%) |  |  |
| Charlson Score |  |  |  |  |  |  |  |  | Chi-square | <0.001 |
| 0-2 | 5,837 (86%) | 218 (68%) | 358 (76%) | 183 (64%) | 298 (82%) | 618 (87%) | 785 (81%) | 3,377 (91%) |  |  |
| 3+ | 974 (14%) | 101 (32%) | 112 (24%) | 102 (36%) | 66 (18%) | 89 (13%) | 188 (19%) | 316 (9%) |  |  |
| Baseline A1c Control |  |  |  |  |  |  |  |  | Chi-square | <0.001 |
| A1c abnormal (>8) pre-SIP | 2,631 (39%) | 153 (48%) | 223 (47%) | 111 (39%) | 161 (44%) | 306 (43%) | 386 (40%) | 1,291 (35%) |  |  |
| Controlled A1c (<8) pre-SIP | 3,307 (49%) | 153 (48%) | 211 (45%) | 161 (56%) | 176 (48%) | 333 (47%) | 490 (50%) | 1,783 (48%) |  |  |
| Missing | 873 (13%) | 13 (4%) | 36 (8%) | 13 (5%) | 27 (7%) | 68 (10%) | 97 (10%) | 619 (17%) |  |  |
| Baseline BP Control |  |  |  |  |  |  |  |  | Chi-square | <0.001 |
| BP <=120/80 | 1,629 (24%) | 84 (26%) | 114 (24%) | 62 (22%) | 81 (22%) | 154 (22%) | 227 (23%) | 907 (25%) |  |  |
| BP <=140/90 | 3,598 (53%) | 169 (53%) | 258 (55%) | 146 (51%) | 205 (56%) | 402 (57%) | 530 (54%) | 1,888 (51%) |  |  |
| BP >140/90 | 1,205 (18%) | 65 (20%) | 82 (17%) | 77 (27%) | 72 (20%) | 126 (18%) | 188 (19%) | 595 (16%) |  |  |
| Missing | 379 (6%) | 1 (0%) | 16 (3%) | 0 (0%) | 6 (2%) | 25 (4%) | 28 (3%) | 303 (8%) |  |  |
| Active MyChart |  |  |  |  |  |  |  |  | Chi-square | <0.001 |
| No | 6,217 (91%) | 279 (87%) | 445 (95%) | 223 (78%) | 301 (83%) | 676 (96%) | 836 (86%) | 3,457 (94%) |  |  |
| Yes | 594 (9%) | 40 (13%) | 25 (5%) | 62 (22%) | 63 (17%) | 31 (4%) | 137 (14%) | 236 (6%) |  |  |

Eth: Ethnicity, Pref: Preference, Eng: English, Non-Eng: Non-English, AA: African American, Un: Unknown, SES: socioeconomic status, A1c: hemoglobin A1c lab result, BP: blood pressure

**Supplementary Note 1**

We framed our cluster findings in a broader context by comparing care trajectories across both health systems, with attention to how pre-pandemic utilization influenced subsequent engagement. We compared the clusters at each health system based on their pre-pandemic utilization behavior, grouping patients as low, moderate, or high utilizers of care prior to the shelter-in-place mandate.

*High Utilizers*

Across both health systems, most clusters of high utilizers returned to pre-pandemic in-person rates, while all adopted elements of digital care, either transitioning to or adding telehealth and/or between-visit care. Clusters UCSF 1, UCSF 2, SFHN 1, and SFHN 2, regained in-person rates approximately one year after the shelter-in-place mandate. Of these, UCSF 1 and SFHN 1 had moderate to high initial uptake of telehealth that eventually trended down and plateaued, while UCSF 2 sustained moderate telehealth uptake over time. In contrast, SFHN 2 had low uptake of telehealth from the start, which was not sustained. All four clusters increased their between-visit care and maintained higher between-visit engagement post-pandemic.

High utilizer clusters UCSF 3 and SFHN 3 did not recover in-person visit rates to pre-pandemic levels and did not increase their between-visit engagement; however, they did increase their telehealth use. At each health system, UCSF 3 and SFHN 3 had the highest initial uptake of telehealth of all clusters, and while UCSF 3 sustained high use over time, SFHN 3 eventually sustained moderate telehealth use.

*Moderate Utilizers*

Moderate utilizers displayed more heterogeneous patterns across health systems. Clusters UCSF 4 and SFHN 4 transitioned to digital care; while in-person visit rates did not recover after the shelter-in-place mandate, both clusters maintained sustained use of telehealth and between-visit care. UCSF 4 sustained consistent moderate uptake of telehealth over time and added more between-visit care. SFHN 4 had high initial uptake of telehealth that declined over time and plateaued with moderate sustained use, as well as adding more between-visit care.

In contrast, clusters UCSF 5 and SFHN 6 lost care as a result of the pandemic, despite moderate pre-pandemic engagement. While neither cluster returned to pre-pandemic in-person rates, they both had moderate initial uptake of telehealth, but use was not sustained over time. Additionally, both clusters exhibited declining between-visit care, with less between-visit engagement than before the pandemic. SFHN 5 similarly did not sustain changes in telehealth or between-visit engagement; however, they returned to pre-pandemic in-person visit rates more quickly than any other cluster.

*Low Utilizers*

Low utilizers represented the largest patient clusters at UCSF and SFHN. Clusters UCSF 7 and SFHN 7 were characterized by loss of care, demonstrated by minimal in-person visits pre-pandemic that did not recover after the shelter-in-place mandate, minimal uptake of telehealth, and consistent low engagement between visits. These two clusters differed in that SFHN 7 demonstrated some engagement with telehealth after the pandemic, but adoption was not sustained, whereas UCSF 7 had low telehealth engagement that did not change much over time. Cluster UCSF 6 demonstrated a different pattern among low utilizers, increasing care engagement through telehealth adoption. This cluster nearly returned to pre-pandemic in-person rates, sustained moderate telehealth uptake, and saw some increase in between-visit care engagement (although this trend declined toward the end of the study period).

**Supplementary Figure 3.**

**Supplementary Figure 3a. State Distribution Plots at UCSF using Optimal Matching**

The state distribution plot shows the proportion of individuals in each care state (in-person, telehealth, and between-visit interactions) at each time point. The x-axis represents time (e.g., months or encounters), and the stacked colored areas indicate the relative frequency of each state. Changes in the height of each colored band over time reflect shifts in the overall composition of care states across the cohort, allowing visualization of dominant modalities and temporal trends in care engagement.

HU: High Utilizer, MU: Moderate Utilizer, LU: Low Utilizer, TH: Telehealth, BVC: Between-Visit Care Interactions

**Supplementary Figure 3b. State Distribution Plots at SFHN using Optimal Matching**

The state distribution plot shows the proportion of individuals in each care state (in-person, telehealth, and between-visit interactions) at each time point. The x-axis represents time (e.g., months or encounters), and the stacked colored areas indicate the relative frequency of each state. Changes in the height of each colored band over time reflect shifts in the overall composition of care states across the cohort, allowing visualization of dominant modalities and temporal trends in care engagement.

HU: High Utilizer, MU: Moderate Utilizer, LU: Low Utilizer, TH: Telehealth, BVC: Between-Visit Care Interactions

**Supplementary Figure 4**

**Supplementary Figure 4a. UCSF Cluster Quality Assessment**

Hubert’s C Index (HC) compares the observed within-cluster dissimilarity to the range of all possible dissimilarities, with lower values indicating better clustering. The average silhouette width (ASW) measures the cohesion and separation of clusters, with higher values reflecting more distinct and well-defined groupings. The Calinski-Harabasz (CH) index assesses the ratio of between-cluster to within-cluster variance, with higher values suggesting greater cluster separation. Together, these metrics provided complementary evidence to identify the most stable and interpretable cluster solution (ranging from k = 2 to k = 10).

**Supplementary Figure 4b. SFHN Cluster Quality Assessment**

Hubert’s C Index (HC) compares the observed within-cluster dissimilarity to the range of all possible dissimilarities, with lower values indicating better clustering. The average silhouette width (ASW) measures the cohesion and separation of clusters, with higher values reflecting more distinct and well-defined groupings. The Calinski-Harabasz (CH) index assesses the ratio of between-cluster to within-cluster variance, with higher values suggesting greater cluster separation. Together, these metrics provided complementary evidence to identify the most stable and interpretable cluster solution (ranging from k = 2 to k = 10).

**Supplementary Figure 5**

**Supplementary Figure 5a. UCSF Mean Number of Quarterly Encounters by Cluster**

The mean quarterly plot shows the mean encounters per patient, per quarter, by modality within each cluster at UCSF for the study period, excluding the Shelter-In-Place period.

HU: High Utilizer, MU: Moderate Utilizer, LU: Low Utilizer, TH: Telehealth, BVC: Between-Visit Care Interactions

**Supplementary Figure 5b. SFHN Mean Number of Quarterly Encounters by Cluster**

The mean quarterly plot shows the mean encounters per patient, per quarter, by modality within each cluster at UCSF for the study period, excluding the Shelter-In-Place period.

HU: High Utilizer, MU: Moderate Utilizer, LU: Low Utilizer, TH: Telehealth, BVC: Between-Visit Care Interactions
